# Targeting mitochondria for the treatment of neurodegenerative diseases

**DOI:** 10.3389/fnins.2026.1835506

**Published:** 2026-07-21

**Authors:** Qian Li, Ming You

**Affiliations:** Center for Cancer Prevention, Houston Methodist Cancer Center, Houston Methodist Research Institute, Houston, TX, United States

**Keywords:** blood–brain Barrie, mitochondrial dysfunction, mitochondrial targeting, nanocarrier delivery system, neurodegenerative diseases

## Abstract

Mitochondria are central regulators of cellular metabolism, redox balance, calcium signaling, and cell survival, making them essential for neuronal function. Because neurons rely heavily on mitochondrial oxidative phosphorylation to meet their high energetic demands, mitochondrial dysfunction has emerged as a key pathogenic driver in major neurodegenerative diseases, including Alzheimer’s disease, Parkinson’s disease, Huntington’s disease, and amyotrophic lateral sclerosis. Defects in mitochondrial bioenergetics, excessive reactive oxygen species production, impaired mitochondrial dynamics, disrupted mitophagy, and dysregulated calcium handling collectively contribute to neuronal damage, synaptic dysfunction, and neuroinflammation. These insights have prompted growing interest in therapeutic strategies that directly target mitochondria to restore organelle homeostasis. Recent advances in chemical biology and nanomedicine have enabled the development of mitochondria-targeted ligands, peptide-based targeting systems, and carrier or nanotechnology-enabled delivery platforms designed to overcome biological barriers and selectively deliver therapeutic cargos to mitochondria within the central nervous system. In this Review, we summarize mitochondrial pathological mechanisms in neurodegenerative diseases and discuss emerging mitochondria-targeted therapeutic strategies, highlighting delivery technologies, therapeutic modalities, and translational challenges. Although most strategies remain at the preclinical or proof-of-principle stage, these advances are beginning to shape a conceptual framework for precision mitochondrial medicine, with the longer-term goal of developing disease-modifying interventions for neurodegenerative disorders.

## Graphical abstract

**Figure:**
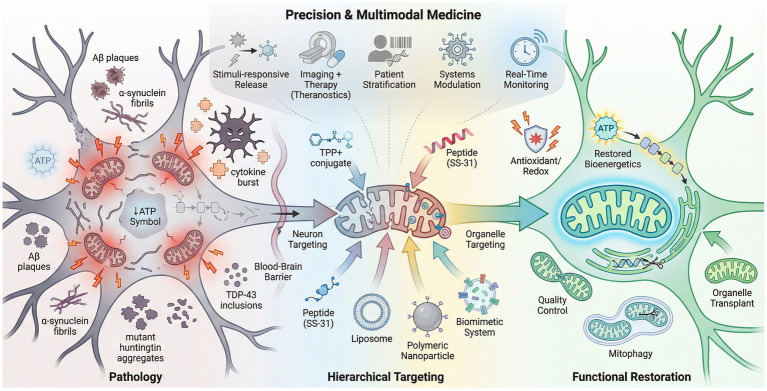
Integrated mitochondrial targeting for neurodegenerative disease.

## 1 Introduction

Mitochondria are central regulators of cellular metabolism and signaling, integrating bioenergetic production with stress adaptation, calcium handling and cell fate determination (Antico et al., 2025; Gray et al., 1999; Lin and Beal, 2006). As the primary site of ATP generation through oxidative phosphorylation, mitochondria sustain the exceptionally high energetic demands of neurons, while simultaneously orchestrating redox signaling, intermediary metabolism and programmed cell death. This multifunctionality positions mitochondrial integrity as a prerequisite for neuronal homeostasis (Amartumur et al., 2024; Lin and Beal, 2006; Pagano Zottola et al., 2025; Rouault, 2013).

Given this central and multifaceted role, mitochondrial integrity is indispensable for cellular and tissue homeostasis. Mitochondrial dysfunction—broadly encompassing impaired oxidative phosphorylation, excessive reactive oxygen species (ROS) accumulation, disrupted mitochondrial dynamics, defective mitophagy and biogenesis, and dysregulated calcium handling—represents a convergent pathological state that culminates in oxidative damage to nucleic acids, proteins, and lipids and ultimately in cellular demise (Kowalczyk et al., 2021; Vasileiou et al., 2019). Accordingly, mitochondrial dysfunction has emerged as a unifying hallmark across a wide spectrum of chronic and degenerative disorders, most prominently neurodegenerative diseases. Neurodegenerative diseases (NDs), including Alzheimer’s disease (AD), Parkinson’s disease (PD), Huntington’s disease (HD), and amyotrophic lateral sclerosis (ALS), are characterized by the progressive and selective loss of neuronal structure and function (Li H. et al., 2021; Liao et al., 2025; Meng K. et al., 2025). Despite their clinical and etiological heterogeneity, accumulating evidence has firmly established mitochondrial dysfunction as a central pathogenic node shared across these disorders. Neurons are among the most energy-demanding cells in the human body and rely almost exclusively on mitochondrial oxidative phosphorylation to sustain their physiological functions (Vercellino and Sazanov, 2022). Strikingly, although the brain accounts for only ~2% of body mass, it consumes nearly 20% of total oxygen, yet is equipped with relatively limited antioxidant defense capacity (Pardo, 2024). This intrinsic metabolic imbalance renders neurons exceptionally vulnerable to mitochondrial perturbations and oxidative stress.

Sustained mitochondrial activity inevitably generates ROS, and failure of detoxification systems initiates a cascade linking mitochondrial damage to neuroinflammation, synaptic dysfunction and cell death pathways (Goate et al., 1991; Vasileiou et al., 2019). This vulnerability is reinforced by intrinsic mitochondrial features: mitochondrial DNA lacks histone protection and exhibits limited repair capacity, enabling age-dependent mutation accumulation that further destabilizes respiratory chain function and establishes self-reinforcing cycles of bioenergetic failure (Mei et al., 2025). At the cellular level, neurodegenerative pathology is therefore characterized by intertwined disturbances in energy metabolism, redox balance, calcium handling, mitochondrial dynamics, biogenesis and mitophagy, frequently exacerbated by mutations in nuclear genes that regulate mitochondrial homeostasis (Wen et al., 2025).

Taken together, mitochondrial dysfunction emerges as a central pathogenic node rather than a secondary consequence of neurodegenerative disease. Consequently, restoring mitochondrial homeostasis has emerged as a mechanistically grounded therapeutic objective. Strategies aimed at modulating mitochondrial redox signaling, rebalancing dynamics, enhancing quality control and improving bioenergetics are increasingly complemented by advances in mitochondria-targeted drug design and nanomedicine-enabled delivery, which offer the possibility of spatiotemporally precise intervention within the central nervous system (Zong et al., 2024). However, achieving effective mitochondrial therapy requires overcoming hierarchical biological barriers, most notably the blood–brain barrier, highlighting the need for integrated targeting frameworks.

In this Review, we examine recent progress in mitochondria-targeted therapeutic strategies for neurodegenerative disease, focusing on targeting ligands, delivery platforms, and emerging biological interventions. We discuss how these approaches address multiscale barriers to mitochondrial access, enable functional restoration of organelle homeostasis, and contribute to an emerging framework for precision mitochondrial medicine.

### 1.1 Literature search strategy

This narrative review was conducted through systematic literature searches of PubMed, Web of Science, and Scopus, covering publications from January 2000 to February 2026, with particular emphasis on studies published after 2015 to reflect recent advances in mitochondrial targeting and nanomedicine. Search terms included combinations of the following: “mitochondria,” “neurodegenerative disease,” “Alzheimer’s disease,” “Parkinson’s disease,” “Huntington’s disease,” “amyotrophic lateral sclerosis,” “mitochondrial dysfunction,” “blood–brain barrier,” “mitochondrial targeting,” “lipophilic cations,” “triphenylphosphonium,” “Szeto–Schiller peptide,” “mitochondria-targeting peptide,” “nanocarrier,” “liposome,” “polymeric nanoparticle,” “biomimetic delivery,” “stimuli-responsive,” “mitochondrial transplantation,” “mitochondrial genome editing,” “mtDNA,” “oxidative stress,” “mitophagy,” “mitochondrial dynamics,” and “neuroinflammation.” Reference lists of key review articles and primary studies were additionally screened to identify seminal works. Inclusion prioritized peer-reviewed original research articles, clinical and preclinical studies, and authoritative reviews with direct mechanistic or therapeutic relevance to the topics covered. Studies were selected to ensure coverage across all four diseases and across the full spectrum of targeting strategies discussed, with preference given to publications demonstrating functional or *in vivo* evidence. Given the breadth of the field, this review is necessarily selective rather than exhaustive; the goal is to provide a conceptually integrated synthesis of representative advances rather than a comprehensive systematic catalog.

Neurodegenerative disorders are characterized by networked mitochondrial dysfunction arising from protein aggregation, oxidative stress, bioenergetic failure and disrupted organelle dynamics. Therapeutic strategies must therefore navigate hierarchical biological barriers—including the blood–brain barrier, cellular membranes and mitochondrial double membranes—using complementary targeting modules such as lipophilic cations, targeting peptides, engineered carriers and biomimetic vesicles. These approaches converge on mitochondria to enable coordinated functional restoration encompassing redox control, bioenergetic recovery, mitochondrial quality maintenance, genome correction and organelle replacement. Emerging precision platforms further integrate stimuli-responsive delivery, theranostic monitoring and patient stratification, supporting spatiotemporal regulation of mitochondrial states. Collectively, this framework illustrates a conceptual shift from single-molecule targeting toward integrated, multi-layered mitochondrial therapeutics that link molecular delivery with organelle repair and systems-level remodeling across neurodegenerative disease. To provide the mechanistic foundation for these therapeutic strategies, we first examine the disease-specific mitochondrial pathology that characterizes each of the four major neurodegenerative disorders addressed in this Review.

## 2 Mitochondrial pathological mechanisms in neurodegenerative diseases

### 2.1 Alzheimer’s disease (AD)

Mitochondrial dysfunction is now recognized as an early and central event in the pathogenesis of AD, preceding overt neurodegeneration and cognitive decline. Rather than representing a secondary consequence of amyloid-*β* (Aβ) and tau pathology, accumulating evidence supports a model in which mitochondrial failure acts as a convergent hub integrating proteotoxic stress, metabolic collapse, redox imbalance, and cell death signaling (Marcos-Rabal et al., 2021; Plascencia-Villa and Perry, 2023).

At the upstream level, AD-associated proteotoxic species directly target mitochondria and compromise their structural and functional integrity (Fernández-Moriano et al., 2015; Oliver and Reddy, 2019). Aβ peptides can translocate into mitochondria through the translocase of the outer membrane (TOM) complex (Roses et al., 2010), where they accumulate within the matrix and interact with mitochondrial proteins such as amyloid-binding alcohol dehydrogenase (ABAD)(Fernández-Moriano et al., 2015) and cyclophilin D (CypD)(Oliver and Reddy, 2019). These interactions enhance ROS production and sensitize the mitochondrial permeability transition pore (mPTP) to pathological opening, thereby lowering the threshold for mitochondrial failure (Bhatia et al., 2022). In parallel, pathological tau disrupts microtubule stability and axonal transport, impairing mitochondrial trafficking to synaptic compartments. This spatial redistribution of mitochondria precedes synaptic degeneration and contributes directly to early synaptic dysfunction, one of the strongest pathological correlates of cognitive decline in AD.

Functionally, mitochondrial targeting by Aβ and tau manifests as a robust bioenergetic deficit. Activities of multiple components of the electron transport chain (ETC), particularly cytochrome c oxidase (complex IV), are significantly reduced, resulting in diminished ATP production and increased electron leakage (Klemmensen et al., 2024). Concomitantly, key enzymes of the tricarboxylic acid (TCA) cycle, including pyruvate dehydrogenase and *α*-ketoglutarate dehydrogenase, exhibit decreased activity (Kang et al., 2021). This combined impairment of oxidative phosphorylation and substrate metabolism establishes a chronic neuronal energy deficit that heightens vulnerability to proteotoxic and oxidative stress.

A direct consequence of impaired electron transport is excessive oxidative stress. Mitochondria act simultaneously as a major source and a principal target of ROS in AD (Misrani et al., 2021). Elevated ROS drives lipid peroxidation, protein oxidation, and nucleic acid damage, while mitochondrial DNA (mtDNA) is particularly susceptible due to its proximity to the respiratory chain and lack of histone protection (Hoekstra et al., 2016). AD brains display increased oxidative lesions and mtDNA mutations (Hirai et al., 2001; Moreira et al., 2006), which further disrupt ETC subunit synthesis and create a self-reinforcing cycle of mitochondrial dysfunction and oxidative damage.

Structural alterations in mitochondrial dynamics further exacerbate these biochemical defects. Physiological mitochondrial function depends on a balance between fission and fusion; however, this equilibrium is markedly disturbed in AD (Reddy and Oliver, 2019). Aβ has been shown to aberrantly activate the fission regulator dynamin-related protein 1 (Drp1), promoting excessive mitochondrial fragmentation. Concurrently, expression of key fusion mediators including mitofusin 1 (MFN1), mitofusin 2 (MFN2), and optic atrophy 1 (OPA1) is reduced in vulnerable regions such as the hippocampus (Choi et al., 2024; Dhapola et al., 2022; Pradeepkiran and Reddy, 2020). This shift toward pathological fragmentation disrupts mitochondrial distribution, connectivity, and functional complementation, thereby amplifying bioenergetic failure.

Mitochondrial calcium handling represents another critical node linking metabolic stress to neuronal death (Singh et al., 2022). Under physiological conditions, mitochondria buffer calcium transients to support synaptic signaling; in AD, this capacity is compromised (Garcia-Casas et al., 2023; Zhang et al., 2022). Aβ-driven dysregulation of calcium homeostasis—arising from aberrant endoplasmic reticulum release and NMDA receptor–mediated influx—leads to mitochondrial calcium overload. Sustained elevation of matrix calcium promotes pathological mPTP opening, collapse of mitochondrial membrane potential, and release of cytochrome c into the cytosol, ultimately activating the caspase-9/3 cascade and engaging the intrinsic apoptotic program (Calvo-Rodriguez et al., 2020; Ott et al., 2008; Supnet and Bezprozvanny, 2010).

**Figure 1:**
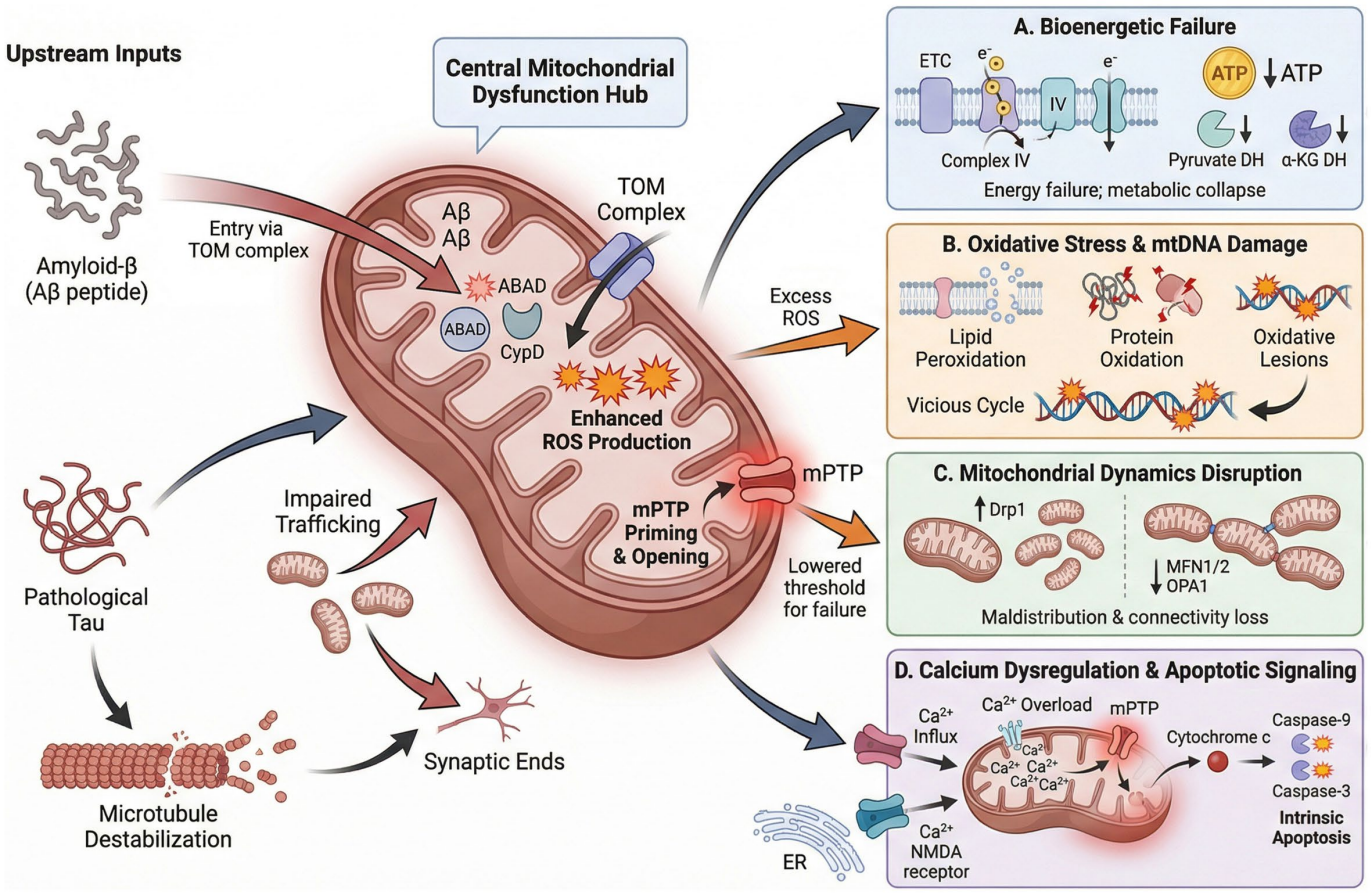
Central role of mitochondrial dysfunction in Alzheimer’s disease. **(A)** Bioenergetic failure. Aβ-mediated mitochondrial impairment reduces electron transport chain activity and key tricarboxylic acid cycle enzymes, leading to ATP depletion and metabolic stress. **(B)** Oxidative stress and mtDNA damage. Defective respiration increases reactive oxygen species production, driving oxidative damage and mitochondrial DNA instability. **(C)** Mitochondrial dynamics disruption. Enhanced fission and reduced fusion promote mitochondrial fragmentation, impairing organelle distribution and connectivity. **(D)** Calcium dysregulation and apoptosis. Mitochondrial calcium overload triggers mPTP opening, cytochrome c release, and activation of intrinsic apoptotic signaling.

Collectively, these findings outline a mechanistic continuum in which proteotoxic mitochondrial targeting leads to bioenergetic failure, oxidative amplification, structural remodeling, and calcium-dependent apoptotic signaling (Figure 1). This integrated cascade positions mitochondrial dysfunction not only as a hallmark of AD pathology but also as a mechanistic bridge linking Aβ and tau pathology to synaptic loss and neuronal degeneration.

### 2.2 Parkinson’s disease (PD)

Parkinson’s disease (PD) is the second most prevalent neurodegenerative disorder worldwide and is neuropathologically characterized by the selective degeneration of dopaminergic neurons in the substantia nigra pars compacta (SNpc) together with the accumulation of Lewy bodies composed primarily of *α*-synuclein in surviving neurons (Poewe et al., 2017; Spillantini et al., 1997; Wong and Krainc, 2017). Increasing evidence indicates that mitochondrial dysfunction represents a central and converging mechanism underlying dopaminergic neurodegeneration in PD (Moon and Paek, 2015).

One of the earliest links between mitochondrial impairment and PD emerged from studies of the neurotoxin MPTP (Langston et al., 1983). In the brain, MPTP is metabolized into MPP^+^, which selectively accumulates in dopaminergic neurons via the dopamine transporter (DAT) and directly inhibits complex I of the mitochondrial ETC (Javitch et al., 1985; Nicklas et al., 1985; Zaichick et al., 2017). This inhibition reduces ATP production and triggers bioenergetic failure. Consistent with this observation, decreased complex I activity has been detected not only in the substantia nigra of sporadic PD patients but also in peripheral tissues such as platelets and skeletal muscle (Chen C. et al., 2023; Schapira et al., 1989). Environmental toxins, including the pesticide rotenone another potent complex I inhibitor can reproduce PD-like pathology, further supporting the etiological relevance of mitochondrial respiratory defects (Betarbet et al., 2000; Hruby and Higuchi-Sanabria, 2025).

Dopaminergic neurons exhibit a particular vulnerability to mitochondrial dysfunction due to their extensive axonal arborization, sustained pacemaking activity, and high metabolic demand (Guzman et al., 2010; Pacelli et al., 2015). In addition, dopamine metabolism itself contributes to oxidative stress: dopamine oxidation generates reactive dopamine quinones and ROS, which interfere with mitochondrial respiration. Elevated iron levels observed in the PD substantia nigra further amplify ROS production through Fenton chemistry (Sian-Hülsmann et al., 2011), creating a vicious cycle of oxidative stress and mitochondrial damage that ultimately promotes neuronal death through apoptosis, necrosis, or ferroptosis (Ayton and Lei, 2014).

Genetic studies of familial PD strongly reinforce the role of mitochondrial quality control pathways in disease pathogenesis (Pickrell and Youle, 2015). Mutations in PINK1 and Parkin disrupt mitophagy, the process responsible for eliminating damaged mitochondria (Cuartero et al., 2018). Under conditions of mitochondrial membrane depolarization, PINK1 accumulates on the outer mitochondrial membrane and recruits the E3 ligase Parkin to initiate autophagic degradation; loss-of-function mutations lead to the accumulation of dysfunctional mitochondria and increased oxidative stress (Youle and Narendra, 2011). *α*-Synuclein, encoded by SNCA, can localize to mitochondrial membranes, where aggregated or mutant forms inhibit complex I activity and interfere with mitochondrial protein import, including through the TOM40 complex. Mutations in LRRK2 are associated with enhanced mitochondrial fission, impaired axonal transport, and suppression of mitophagy, whereas loss of DJ-1 an oxidative stress sensor results in mitochondrial fragmentation and functional deficits (Nakamura et al., 2008; Ryan et al., 2018).

Alterations in mitochondrial dynamics and trafficking further contribute to PD pathology. Mitochondria continuously undergo fusion (mediated by Mfn1/2 and OPA1) and fission (regulated by Drp1 and Fis1) to maintain functional integrity (Chen R. et al., 2023; Ryan et al., 2018). Excessive activation of Drp1 leads to mitochondrial fragmentation, reduced bioenergetic efficiency, and increased susceptibility to stress (Youle and van der Bliek, 2012). Moreover, defects in mitochondrial transport along axons, an early feature of PD, limit energy supply at synaptic terminals and impair neurotransmitter release, exacerbating neuronal dysfunction (Sheng and Cai, 2012).

Additional layers of mitochondrial pathology include mtDNA damage and calcium dysregulation. Elevated levels of mtDNA deletions and point mutations have been reported in the substantia nigra of PD patients, compromising respiratory chain integrity (Kraytsberg et al., 2006). Loss of mitochondrial membrane potential also impairs calcium buffering capacity, resulting in intracellular calcium overload (Rizzuto et al., 2012). The combined effects of calcium dysregulation and oxidative stress promote opening of the mitochondrial permeability transition pore (mPTP), leading to cytochrome c release and activation of intrinsic apoptotic pathways (Giorgi et al., 2018).

**Figure 2:**
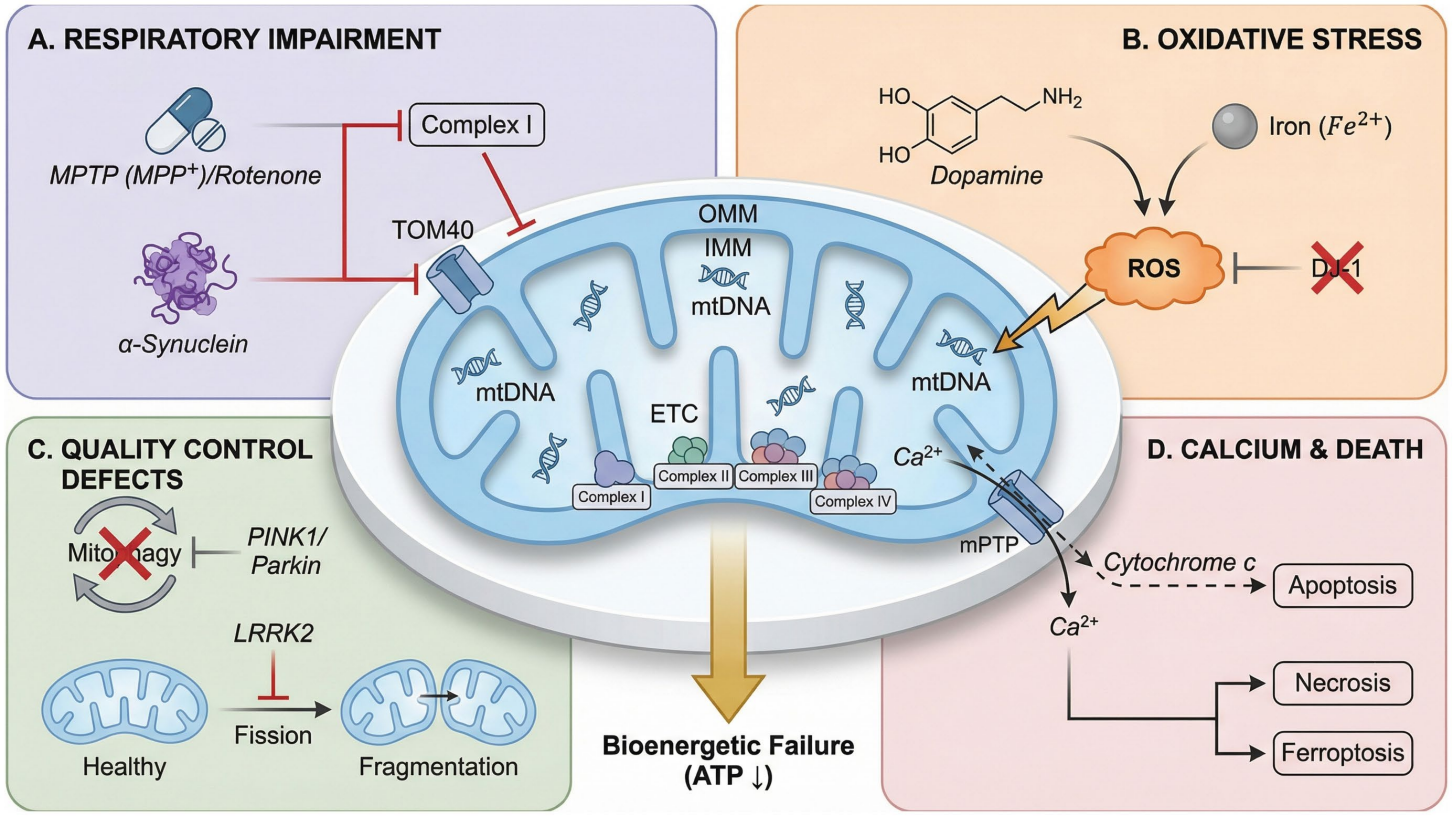
Mitochondrial pathological cascade in Parkinson’s disease. **(A)** Mitochondrial respiratory impairment. Neurotoxins such as MPTP/MPP^+^ and rotenone inhibit complex I of the electron transport chain, while mitochondrial α-synuclein further disrupts respiration and protein import. **(B)** Oxidative stress amplification. Dopamine oxidation and iron accumulation enhance reactive oxygen species production, promoting mitochondrial damage and neuronal vulnerability. **(C)** Defective mitochondrial quality control and dynamics. Mutations in PINK1, Parkin, LRRK2 and DJ-1 impair mitophagy and promote excessive mitochondrial fission, leading to fragmentation and trafficking deficits. **(D)** Calcium dysregulation and neuronal death. Loss of mitochondrial membrane potential reduces calcium buffering, facilitating mPTP opening, cytochrome c release and activation of intrinsic apoptotic signaling.

In conclusion, mitochondrial respiratory defects, oxidative stress, impaired quality control, altered dynamics, and genomic instability form an interconnected network driving dopaminergic neurodegeneration in PD (Figure 2). These findings position mitochondrial pathways not only as key mechanistic contributors to disease progression but also as promising therapeutic targets for disease-modifying interventions.

### 2.3 Huntington’s disease (HD)

Huntington’s disease (HD) is an autosomal dominant neurodegenerative disorder caused by an expanded CAG repeat in the HTT gene, leading to the production of mutant huntingtin protein (mHTT)(Bates et al., 2015; MacDonald et al., 1993). Neuropathologically, HD is characterized by the progressive loss of medium spiny neurons in the striatum, accompanied by widespread metabolic disturbances (Cowan and Raymond, 2006). Among the cellular pathways implicated in HD, mitochondrial dysfunction has emerged as a central contributor to neuronal vulnerability and disease progression (Jurcau and Jurcau, 2023).

Defects in the mitochondrial respiratory chain and bioenergetic metabolism represent one of the most consistent findings in HD. Studies of patient brain tissue, particularly within the striatum, have demonstrated reduced activity of respiratory chain complexes II and III, with milder impairment of complex IV (Burtscher et al., 2020). The selective involvement of complex II is especially notable, as systemic administration of the mitochondrial toxin 3-nitropropionic acid (3-NP), a complex II inhibitor, reproduces HD-like striatal degeneration and motor phenotypes in experimental models (Brouillet et al., 1995). These respiratory defects lead to decreased ATP production, reduced phosphocreatine levels, and lactate accumulation, collectively reflecting a state of neuronal bioenergetic crisis (Martínez Lozada et al., 2024).

A key mechanistic driver of mitochondrial pathology in HD is the direct interaction between mHTT and mitochondrial membranes. Mutant huntingtin and its aggregates can localize to the outer mitochondrial membrane, where they promote abnormal opening of the mPTP, resulting in membrane depolarization and mitochondrial swelling (Carmo et al., 2018; Yablonska et al., 2019). In addition, mHTT interferes with mitochondrial protein import, limiting the entry of nuclear-encoded mitochondrial proteins and further compromising organelle function (Yablonska et al., 2019; Yano et al., 2014).

Calcium dysregulation and excitotoxicity provide another major axis of mitochondrial dysfunction in HD. Mitochondria derived from HD neurons display increased sensitivity to calcium, undergoing rapid depolarization in response to calcium challenge (Choo et al., 2004). This impaired buffering capacity, combined with excessive activation of glutamatergic receptors such as NMDA receptors, drives excitotoxic stress and contributes to the selective degeneration of medium spiny neurons (Heng et al., 2009).

Oxidative stress and mtDNA damage further exacerbate mitochondrial impairment. Dysfunctional mitochondria generate elevated levels of ROS, which oxidatively damage proteins, lipids, and nucleic acids (Barnham et al., 2004; Palma et al., 2024). Iron–sulfur cluster–containing enzymes such as aconitase are particularly vulnerable, and reduced aconitase activity has been observed in the striatum and cortex of HD patients (Ayala-Peña, 2013). Because mtDNA lacks histone protection and resides near the respiratory chain—the primary source of ROS—mtDNA mutations and deletions accumulate with disease progression, reinforcing a cycle of mitochondrial deterioration (Kujoth et al., 2005).

Altered mitochondrial dynamics and impaired axonal transport are also prominent features of HD pathology. Excessive mitochondrial fragmentation has been linked to the ability of mHTT to interact with the fission protein Drp1 and enhance its GTPase activity, thereby promoting mitochondrial division. Concurrently, levels of fusion proteins including Mfn1/2 and OPA1 are reduced in affected tissues (Shirendeb et al., 2011). Aggregated mHTT can additionally obstruct axonal transport pathways, limiting mitochondrial delivery to synaptic terminals and leading to synaptic energy failure and functional decline (Cepeda and Levine, 2022; White et al., 2015).

Mitochondrial biogenesis and mitophagy are likewise disrupted in HD. Mutant huntingtin suppress the expression of PGC-1α, a master regulator of mitochondrial biogenesis, through interference with transcriptional regulatory networks, thereby limiting the generation of new mitochondria (Johri et al., 2013; Joshi et al., 2025). Mitophagy is also compromised: whereas wild-type huntingtin participates in autophagic scaffolding, mHTT impairs the recognition and sequestration of damaged mitochondria, allowing dysfunctional organelles to accumulate and promote cellular stress (Rui et al., 2015).

These mitochondrial abnormalities ultimately converge on activation of intrinsic apoptotic pathways. HD models demonstrate increased expression of pro-apoptotic proteins such as Bax and Bak, along with dysregulation of anti-apoptotic Bcl-2 family members (Portera-Cailliau et al., 1995). This imbalance facilitates the release of cytochrome c and Smac/DIABLO from mitochondria, leading to activation of Caspase-9 and Caspase-3 and driving programmed neuronal death (Mejia and Friedlander, 2001).

**Figure 3:**
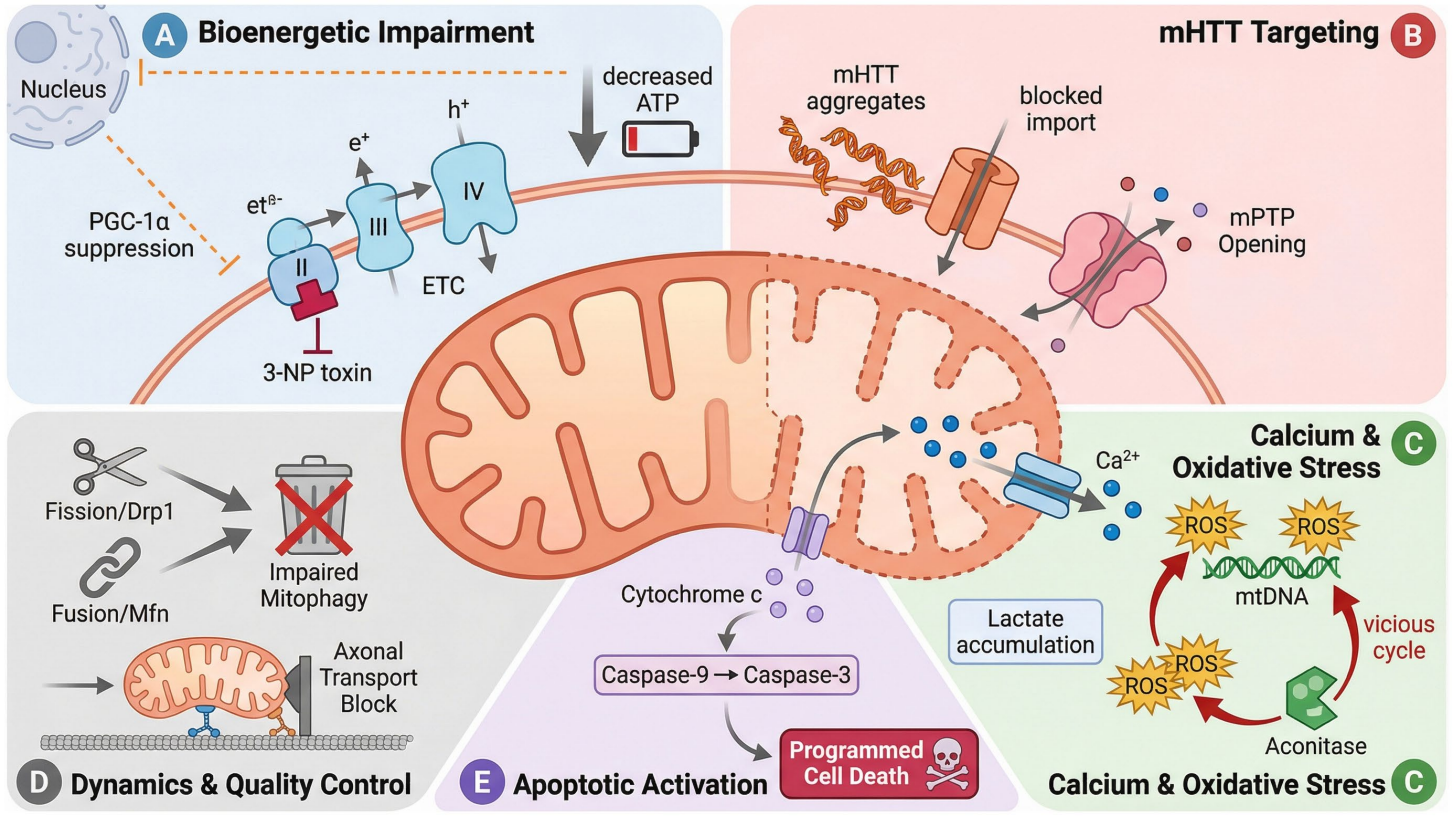
Mitochondrial pathological mechanisms in Huntington’s disease. **(A)** Bioenergetic impairment. Reduced activity of respiratory chain complexes II and III disrupts ATP production and metabolic homeostasis. **(B)** mHTT–mitochondria interaction. Mutant huntingtin localizes to mitochondrial membranes, promotes mPTP opening and interferes with mitochondrial protein import. **(C)** Calcium dysregulation and oxidative stress. Increased mitochondrial calcium sensitivity and ROS production drive excitotoxic injury and mtDNA damage. **(D)** Dynamics and quality control failure. Enhanced Drp1-mediated fission, impaired fusion, suppressed biogenesis and defective mitophagy lead to mitochondrial fragmentation and accumulation of dysfunctional organelles. **(E)** Apoptotic activation. Mitochondrial damage triggers cytochrome c release and caspase-dependent neuronal death.

Taken together, respiratory chain impairment, direct mHTT–mitochondria interactions, calcium dysregulation, oxidative damage, altered dynamics, and defective quality control form an integrated network of mitochondrial pathology in HD (Figure 3). These findings highlight mitochondrial pathways as central mediators of disease progression and underscore their potential as targets for therapeutic intervention.

### 2.4 Amyotrophic lateral sclerosis (ALS)

Amyotrophic lateral sclerosis (ALS) is a progressive neurodegenerative disorder characterized by selective degeneration of upper and lower motor neurons, leading to muscle weakness, atrophy and paralysis (Cauchi and Tosolini, 2025; Hardiman et al., 2017). Despite etiological heterogeneity, mitochondrial dysfunction has emerged as a central and converging mechanism underlying motor neuron vulnerability (Muyderman and Chen, 2014).

A key initiating event involves the mitochondrial localization of disease-associated proteins. Mutant superoxide dismutase 1 (SOD1) accumulates within mitochondria, disrupting membrane integrity and interfering with voltage-dependent anion channel 1 (VDAC1), thereby impairing metabolite exchange (Israelson et al., 2010; Takeuchi et al., 2002). Similarly, TAR DNA-binding protein 43 (TDP-43) aggregates translocate into mitochondria and suppress mitochondrial protein translation, producing structural and functional deficits that represent early drivers of mitochondrial failure (Gao et al., 2019).

Given the exceptionally high energetic demands of motor neurons, mitochondrial respiratory impairment has profound consequences. Reduced electron transport chain activity and ATP production have been consistently observed in ALS patient tissues, establishing a chronic bioenergetic deficit that compromises neuronal homeostasis (Muyderman and Chen, 2014; Ovsepian et al., 2024). This metabolic vulnerability is reinforced by oxidative stress, as dysfunctional mitochondria generate elevated ROS and accumulate mtDNA damage, further destabilizing respiratory function (Yang et al., 2008).

Alterations in mitochondrial dynamics and transport amplify these defects. ALS is associated with excessive mitochondrial fragmentation, characterized by increased fission regulators and reduced fusion proteins, alongside impaired microtubule-dependent trafficking (Cappello and Francolini, 2017). The resulting redistribution of mitochondria—enrichment in neuronal soma and depletion at distal axons—contributes to neuromuscular junction failure and progressive denervation (Dadon-Nachum et al., 2011).

Mitochondrial quality control pathways are also disrupted. Mutations in OPTN, SQSTM1 and VCP interfere with PINK1/Parkin-mediated mitophagy, allowing damaged mitochondria to accumulate and sustain cellular stress (Nakazawa et al., 2016). In parallel, defective endoplasmic reticulum–mitochondria communication at mitochondria-associated membranes (MAMs), driven by VAPB and CHCHD10 mutations, perturbs calcium signalling and mitochondrial ultrastructure (Smith et al., 2019).

**Figure 4:**
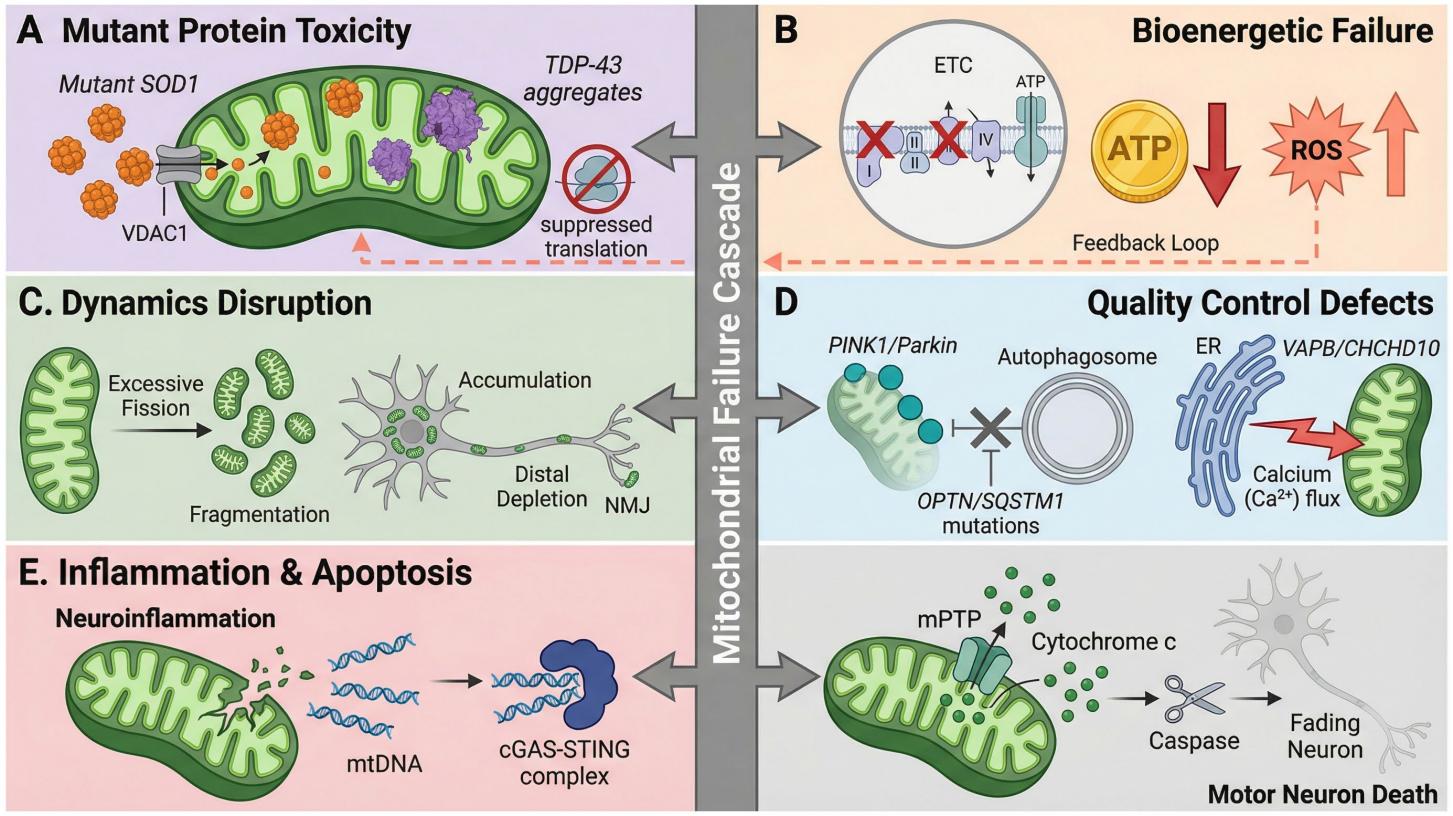
Mitochondrial pathological mechanisms in amyotrophic lateral sclerosis. **(A)** Mutant protein mitochondrial toxicity. SOD1 and TDP-43 accumulate in mitochondria, disrupting membrane integrity and mitochondrial protein translation. **(B)** Bioenergetic failure and oxidative stress. Impaired respiratory chain activity reduces ATP production and increases reactive oxygen species and mtDNA damage. **(C)** Dynamics and transport disruption. Excessive mitochondrial fragmentation and defective axonal trafficking lead to mitochondrial redistribution and neuromuscular junction failure. **(D)** Quality control and organelle crosstalk defects. Impaired mitophagy and disrupted ER–mitochondria communication alters calcium signalling and mitochondrial structure. **(E)** Inflammation and apoptotic signalling. mtDNA release activates cGAS–STING–mediated neuroinflammation, while mPTP opening triggers cytochrome c release and motor neuron death.

Ultimately, these interconnected processes converge on inflammatory and apoptotic signaling. Release of mitochondrial DNA can activate cGAS–STING-dependent neuroinflammation, while loss of membrane potential and permeability transition pore opening trigger cytochrome c release and caspase-mediated motor neuron death (Hu and Shu, 2023). Collectively, mutant protein toxicity, metabolic failure, impaired dynamics and defective quality control form an integrated mitochondrial cascade that underpins ALS progression and highlights mitochondria as a central therapeutic target (Figure 4).

Across Alzheimer’s disease, Parkinson’s disease, Huntington’s disease, and amyotrophic lateral sclerosis, mitochondrial dysfunction emerges as a shared pathological hub linking diverse upstream insults to convergent neuronal outcomes (Table 1). Despite disease-specific triggers—such as Aβ/tau, *α*-synuclein, mutant huntingtin, or SOD1/TDP-43—these disorders consistently exhibit impaired bioenergetics, oxidative stress, disrupted mitochondrial dynamics and trafficking, defective quality control, and calcium dysregulation, ultimately promoting neuroinflammation and cell death. This cross-disease convergence highlights mitochondria not only as vulnerable targets of proteotoxic stress but also as active integrators that translate heterogeneous molecular pathology into common neurodegenerative cascades, supporting mitochondria-centered therapeutic strategies.

**Table 1:** Mitochondrial pathological mechanisms across AD, PD, HD, and ALS.

Mechanistic layer	Alzheimer’s disease (AD)	Parkinson’s disease (PD)	Huntington’s disease (HD)	Amyotrophic lateral sclerosis (ALS)
Proteotoxic mitochondrial targeting	Aβ import via TOM; interaction with ABAD and CypD; tau impairs trafficking	α-Synuclein localizes to mitochondria; inhibits complex I; import defects	mHTT associates with mitochondrial membranes; interferes with protein import	Mutant SOD1 accumulates in mitochondria; TDP-43 suppresses mitochondrial translation
Respiratory chain/bioenergetics defects	ETC reduction (notably complex IV); TCA enzyme decline	Complex I inhibition (MPTP/rotenone models)	Complex II/III impairment; ATP depletion	Reduced respiratory capacity; ATP insufficiency in motor neurons
Oxidative stress and mtDNA damage	ROS amplification; mtDNA mutations	Dopamine oxidation + iron-driven ROS; mtDNA damage	ROS elevation; aconitase sensitivity; mtDNA instability	Elevated ROS; mtDNA damage in motor cortex and spinal cord
Mitochondrial dynamics dysregulation	Drp1 activation; MFN1/2 and OPA1 reduction; fragmentation	Excess fission; impaired transport	Drp1 hyperactivation via mHTT; reduced fusion	Increased Drp1/Fis1; decreased fusion proteins
Axonal transport defects	Tau-mediated trafficking impairment	Early mitochondrial transport deficits	mHTT blocks axonal transport	Mitochondria accumulate in soma; depleted at NMJs
Calcium dysregulation	ER/NMDA-driven mitochondrial Ca^2+^ overload	Membrane potential loss impairs buffering	Increased Ca^2+^ sensitivity; excitotoxicity	MAM disruption alters Ca^2+^ signaling
Quality control failure (mitophagy/biogenesis)	Mitophagy inefficiency (emerging)	PINK1/Parkin mutations impair mitophagy	PGC-1α suppression; defective mitophagy	OPTN/SQSTM1/VCP mutations impair mitophagy
Inflammatory signaling	mtDNA/ROS activate NLRP3 and STING	Neuroinflammation secondary to mitochondrial stress	Increasing evidence but less defined	mtDNA release activates cGAS–STING
Cell death pathways	mPTP opening → caspase cascade	Apoptosis, ferroptosis	Bax/Bak activation; caspase signaling	Apoptosis + neuroinflammation-driven degeneration

While Table 1 highlights mechanistic convergence across neurodegenerative diseases, important disease-specific distinctions must be acknowledged. In AD, mitochondrial dysfunction manifests as an early pathological event that precedes overt neurodegeneration, suggesting a broad therapeutic window but also raising questions about causality versus consequence relative to amyloid and tau pathology. In PD, genetic evidence from PINK1, Parkin and LRRK2 mutations establishes mitochondrial quality control failure as a primary causal driver in familial forms, though the temporal relationship in sporadic disease remains less defined. In HD, mitochondrial impairment is largely downstream of mHTT toxicity and may therefore represent a secondary amplifier rather than an initiating event, which has implications for the positioning of mitochondrial therapies within a broader treatment strategy. In ALS, mitochondrial pathology is heterogeneous across genetic subtypes—SOD1, TDP-43 and FUS mutations engage mitochondria through distinct mechanisms—and the rapidly progressive disease course narrows the effective intervention window considerably. Furthermore, the selective neuronal populations affected in each disease—hippocampal and cortical neurons in AD, dopaminergic neurons in PD, striatal medium spiny neurons in HD, and upper and lower motor neurons in ALS—differ substantially in their metabolic demands, mitochondrial morphology and susceptibility to specific insults, underscoring that mitochondrial targeting strategies may need to be tailored rather than uniformly applied across diseases.

Collectively, these cross-disease mitochondrial abnormalities establish a mechanistic rationale for therapeutic strategies that directly target mitochondrial dysfunction. However, effective intervention requires not only modulation of mitochondrial pathways, but also the ability to overcome multiple biological barriers and selectively access mitochondria within vulnerable neuronal populations. These challenges have driven the development of diverse mitochondrial targeting ligands and molecular delivery strategies.

## 3 Mitochondrial targeting ligands and molecular strategies

**Figure 5:**
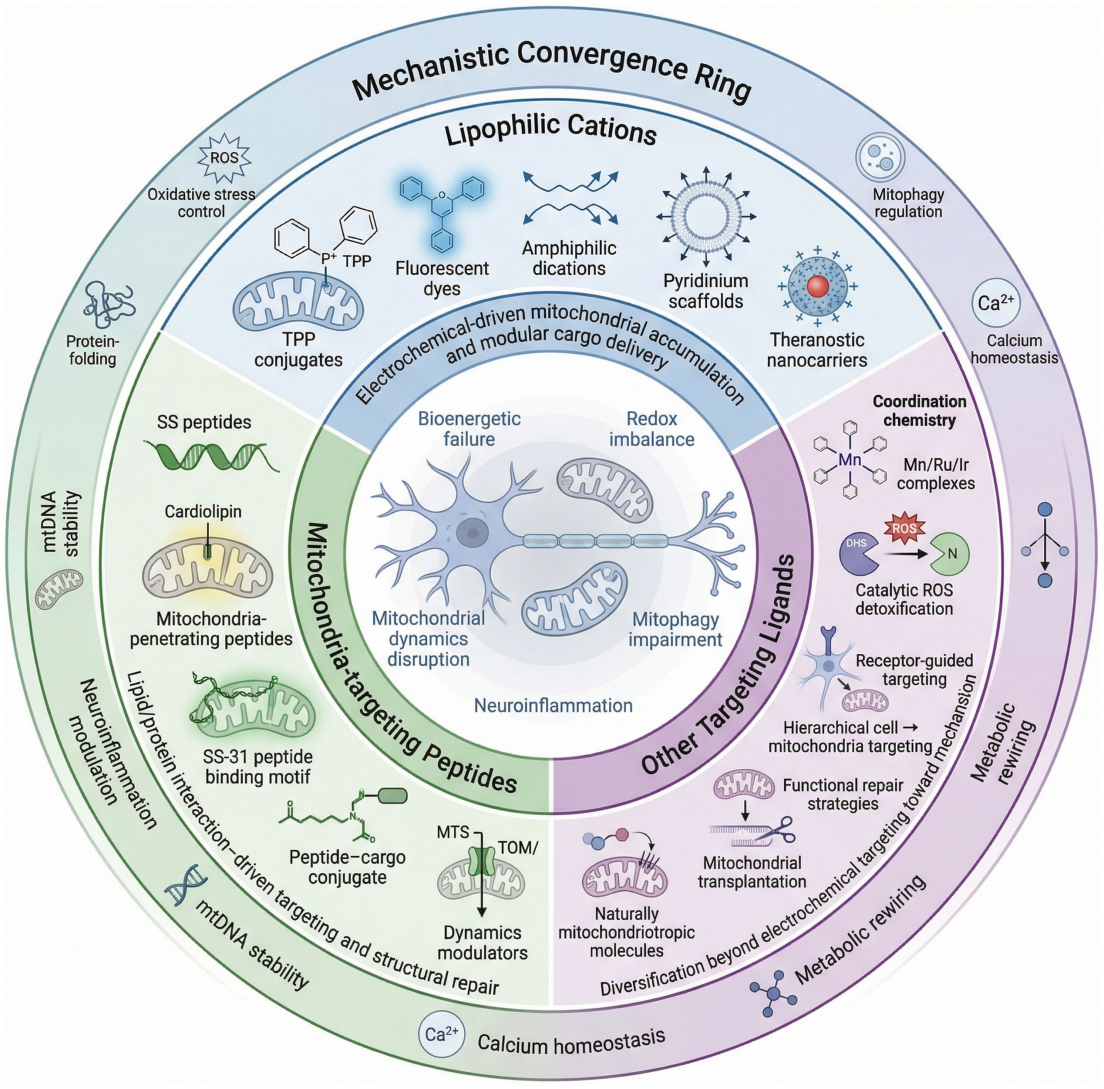
Landscape of mitochondrial targeting ligands and molecular strategies in neurodegenerative disease. Mitochondrial targeting has evolved into a hierarchical and multimodal design framework that integrates chemically distinct ligand classes with convergent biological functions. Lipophilic cations exploit the mitochondrial membrane potential to drive electrochemical accumulation and modular cargo delivery, encompassing triphenylphosphonium conjugates, fluorescent dyes, amphiphilic dications, pyridinium scaffolds and theranostic nanocarriers. Mitochondria-targeting peptides engage lipid–protein interactions within mitochondrial membranes and import pathways, including Szeto–Schiller peptides, mitochondria-penetrating peptides, peptide–cargo conjugates and mitochondrial targeting sequences that enable structural stabilization and functional repair. Complementary targeting ligands expand this landscape beyond electrochemical accumulation through coordination chemistry, receptor-guided recognition and functional restoration strategies such as mitochondrial transplantation and genome-directed intervention. Across these modalities, diverse molecular approaches converge on shared mechanistic endpoints—including bioenergetic regulation, redox homeostasis, proteostasis, mitophagy control, calcium signalling and neuroinflammation—highlighting a transition from organelle localization toward integrated mitochondrial therapeutic architectures.

Mitochondrial targeting has emerged as a central design paradigm in the development of disease-modifying strategies for neurodegenerative disorders. Rather than relying on a single class of ligands, the field now encompasses a broad spectrum of molecular and supramolecular approaches that differ in targeting mechanism, cargo compatibility and functional scope. As illustrated in Figure 5, contemporary mitochondrial delivery frameworks can be broadly organized into three interrelated modalities: electrochemically driven small-molecule targeting exemplified by lipophilic cations, biologically inspired peptide-based systems that engage mitochondrial membranes and import machinery, and a rapidly expanding set of alternative ligands that incorporate coordination chemistry, receptor-guided recognition and functional repair strategies. This conceptual landscape reflects a transition from simple organelle localization toward integrated therapeutic architectures capable of simultaneously modulating mitochondrial bioenergetics, redox homeostasis, proteostasis and inflammatory signaling.

### 3.1 Lipophilic cations

Lipophilic cations represent one of the most extensively investigated molecular strategies for mitochondrial targeting, owing to their ability to exploit the distinctive electrochemical landscape of mitochondria (Zielonka et al., 2017). The large negative membrane potential (ΔΨ_m_) across the inner mitochondrial membrane, generated by proton pumping during oxidative phosphorylation, creates a powerful driving force for the accumulation of positively charged, hydrophobic molecules (Kopecka, 2022; Sakamuru et al., 2016; Zielonka et al., 2017). Compared with the relatively modest plasma membrane potential, this electrochemical gradient enables lipophilic cations with delocalized charge to diffuse across biological membranes and concentrate electrophoretically within the mitochondrial matrix, often reaching orders of magnitude higher levels than in the cytosol (Džajić et al., 2025).

Among these ligands, triphenylphosphonium (TPP^+^) has emerged as the prototypical scaffold, demonstrating how simple chemical conjugation can reshape mitochondrial pharmacology in neurodegenerative disease (Zielonka et al., 2017). TPP^+^-linked quinone derivatives such as MitoQ and SkQ1 leverage reversible redox cycling to attenuate mitochondrial oxidative stress while stabilizing electron transport chain activity, whereas nitroxide-based constructs including MitoTEMPO provide catalytic buffering of reactive oxygen species and iron-mediated toxicity (Hu and Li, 2016). Lipid-targeted antioxidants such as MitoVitE emphasize membrane preservation, particularly cardiolipin integrity, while thiol-conjugated approaches including MitoGSH and MitoNAC reinforce endogenous redox networks (Ramalingam et al., 2021; Zielonka et al., 2017). Collectively, these examples illustrate the modularity of the TPP^+^ scaffold, enabling diverse functional cargos to interface with mitochondrial bioenergetics, redox balance and structural stability.

Beyond TPP^+^, a broader chemical space of lipophilic cations has expanded mitochondrial targeting strategies. Fluorescent dye scaffolds such as rhodamine and cyanine derivatives provide intrinsic imaging capability alongside mitochondrial accumulation, facilitating theranostic applications in neurodegenerative disease (Džajić et al., 2025; Johnson et al., 1980). Amphiphilic dications such as dequalinium introduce self-assembling delivery platforms capable of transporting nucleic acids and small molecules into mitochondria, bridging small-molecule targeting and nanocarrier design (Jeena et al., 2020; Kopecka, 2022; Weissig, 2015). Similarly, pyridinium-based systems and cationic natural-product derivatives, including berberine analogs, offer electronically tunable frameworks that integrate mitochondrial targeting with sensing, photomodulation or metabolic regulation (Jiao et al., 2023; Murphy and Hartley, 2018). These expanded scaffolds highlight a conceptual shift in which lipophilic cations serve not only as delivery tags but also as functional components within multi-modal therapeutic architectures.

Despite their versatility, lipophilic cation strategies share inherent constraints. Their accumulation remains tightly coupled to membrane potential, biasing delivery toward relatively polarized mitochondria and limiting access to severely dysfunctional organelles (Fields et al., 2023). This membrane potential dependence creates a fundamental therapeutic paradox: the progressive mitochondrial depolarization that characterizes severely affected neurons in AD, PD and ALS reduces TPP^+^-driven accumulation precisely where intervention is most needed. Yet most preclinical efficacy studies employ healthy or mildly stressed neurons that retain near-physiological membrane potentials, potentially overestimating the targeting efficiency achievable in advanced disease. Systematic evaluation of TPP^+^ delivery performance across disease-relevant, depolarized mitochondrial conditions remains an important unmet need, as does the development of membrane-potential-independent targeting strategies capable of functioning in severely dysfunctional organelles. High local enrichment can perturb membrane integrity and oxidative phosphorylation, narrowing therapeutic windows during chronic treatment, while the physicochemical requirements for membrane permeation restrict the range of deliverable cargos (Kulkarni et al., 2021; Xu et al., 2025). These limitations have driven increasing interest in hybrid approaches that integrate lipophilic cation targeting with carrier-based delivery.

Accordingly, the field is progressively converging on hierarchical and multimodal mitochondrial delivery frameworks. Incorporating lipophilic cations—including TPP^+^ and alternative scaffolds—into polymeric nanoparticles, liposomes and biomimetic vesicles enables controlled release, improved pharmacokinetics and reduced concentration-dependent mitochondrial perturbation (Table 2). Importantly, such platforms allow simultaneous modulation of multiple disease-relevant processes, including oxidative stress, proteostasis, mitophagy and neuroinflammation, while supporting imaging-enabled patient stratification. In this emerging landscape, lipophilic cations are increasingly positioned not as standalone solutions but as foundational elements within integrated mitochondrial therapeutic strategies for neurodegenerative disorders. Compared with peptide-based systems, lipophilic cations achieve higher mitochondrial accumulation in polarized mitochondria but show inferior BBB penetration without carrier assistance and are more susceptible to concentration-dependent toxicity.

**Table 2:** Representative lipophilic cations compounds: chemical features and neurodegenerative-disease fit.

Compound (TPP^+^ conjugate)	Cargo class/core chemistry	Primary mitochondrial action	Chemical advantages	Chemical liabilities	Best-fit NDD context (conceptual)	Key translational bottlenecks
MitoQ (Murphy and Smith, 2007; Smith et al., 2012)	Quinone (ubiquinone/ubiquinol cycling)	Redox cycling; lipid peroxidation suppression	Sustained activity via ETC-mediated reduction; strong IMM association	Context-dependent pro-oxidant risk at high accumulation; ΔΨm dependence	AD/PD where ROS and ETC stress are early features	BBB exposure; chronic dosing safety; stage dependence
SkQ1 (Jiang et al., 2018; Skulachev et al., 2009)	Plastoquinone derivative	Cardiolipin-associated antioxidant protection	High membrane/lipid targeting; cristae stabilization potential	High lipophilicity may increase nonspecific membrane perturbation	AD/aging-related synaptic mitochondrial fragility; inflammation-linked injury	Long-term safety; distribution control (Zielonka et al., 2017)
MitoTEMPO/MitoTEMPOL (Dikalov and Harrison, 2014; Trnka et al., 2008)	Nitroxide radical (SOD mimetic)	Catalytic ROS scavenging; oxidative stress attenuation	Catalytic-like ROS handling; strong fit for dopaminergic oxidative stress	Radical chemistry can have off-target reactivity; PK variability	PD (dopamine/iron-driven ROS burden)	Exposure/retention in brain; dose-limited toxicity
MitoVitE (McCormick et al., 2016; Plecitá-Hlavatá et al., 2009; Smith et al., 2003)	α-Tocopherol (lipid antioxidant)	Inhibits lipid peroxidation in membranes	Strong membrane protection; synergy with ROS reduction	Very lipophilic → nonspecific membrane binding; accumulation toxicity risk	AD where lipid peroxidation/cardiolipin damage contributes to synapse loss	Safety window; formulation challenges
MitoGSH (Kezic et al., 2016; Liu Y. et al., 2023; Marí et al., 2020)	Glutathione-linked thiol system	Enhances endogenous redox buffering	Aligns with native antioxidant networks; complements catalytic scavengers	Thiol instability/metabolism; limited persistence	Broad NDDs with redox imbalance (AD/PD/ALS)	Bioavailability; stability; sustained efficacy
MitoNAC (Cheng et al., 2023; Vilas-Boas et al., 2023)	N-acetylcysteine-linked thiol	Supports GSH synthesis; redox buffering	Simple chemistry; boosts endogenous defense	Similar thiol limitations; dosing may be high	Broad NDDs; potentially ALS where oxidative stress is prominent	BBB penetration; chronic regimen practicality
Mito-metformin (Ay et al., 2024; Loan et al., 2024)	Biguanide (metabolic modulator)	Bioenergetics/biogenesis signalling modulation	Repurposing logic; targets metabolic component of NDDs	Risk of mitochondrial over-inhibition if excessive; ΔΨm dependence	PD/aging-related mitochondrial biogenesis deficits	Mechanism complexity; tolerability and dose optimization
Rhodamine derivatives (Chauhan et al., 2022; Silachev et al., 2015)	Delocalized aromatic cation dye	Mitochondrial imaging; carrier-assisted antioxidant delivery	Strong membrane permeability; intrinsic fluorescence; tunable chemistry	Phototoxicity risk; limited therapeutic potency alone	AD/PD mitochondrial dysfunction mapping; theranostics	Stability; toxicity; limited drug payload capacity
Dequalinium (DQA)/DQAsomes (Mursaleen et al., 2023; Yue Zhang et al., 2021)	Dicationic amphiphile vesicle-forming system	Gene and small-molecule mitochondrial delivery	Self-assembly; macromolecule delivery potential; dual targeting	Complex PK; cellular toxicity at high dose	Emerging for neurodegeneration gene modulation; mitochondrial repair	Scale-up; BBB delivery; safety profile
Pyridinium-based cations (Xu et al., 2022; Zielonka et al., 2017)	Aromatic heterocycle cation	ROS sensing; mitochondrial phototherapy; cargo conjugation	Electronic tunability; optical activity; modular synthesis	Potential off-target reactivity; limited clinical experience	AD imaging; anti-aggregation strategies	Translational validation; pharmacokinetics
Cyanine dyes (mitochondria-targeted) (Kim J. et al., 2023; Yan et al., 2024)	Extended conjugated cationic dye	Imaging; photothermal/photodynamic modulation	Strong mitochondrial accumulation; BBB penetration; theranostics	Phototoxicity; metabolic instability	Neuroinflammation imaging; mitochondrial dysfunction monitoring	Safety; light-based therapy limitations
Berberine derivatives (cationic alkaloids)(Aryal et al., 2022; Yuan et al., 2019)	Planar bioactive cation scaffold	Modulation of mitochondrial metabolism and ROS	Natural product origin; BBB penetration; multi-target activity	Off-target signaling; variable mitochondrial specificity	AD/PD metabolic dysfunction; neuroinflammation	Dose optimization; specificity

### 3.2 Mitochondria-targeting peptides

Mitochondria-targeting peptides (MTPs) represent a biologically inspired alternative to small-molecule lipophilic cations for mitochondrial delivery (Jiang et al., 2021; Zielonka et al., 2017). These peptides are typically composed of alternating aromatic residues (phenylalanine or tyrosine) and cationic residues (lysine or arginine), enabling membrane interaction, electrostatic attraction and selective mitochondrial accumulation (Mitchell et al., 2020; Nguyen et al., 2024; Nguyen et al., 2025). Compared with classical lipophilic cations such as TPP^+^, peptide-based targeting offers improved tunability, favorable biocompatibility and reduced mitochondrial depolarization at pharmacologically relevant concentrations (Zielonka et al., 2017). Importantly, several MTPs exhibit efficient cellular uptake and can engage mitochondrial membranes through mechanisms that are partially independent of membrane potential (Tamucci et al., 2023).

Among these systems, the Szeto–Schiller (SS) peptides are the most extensively studied. SS peptides share a conserved motif of alternating aromatic and basic residues, with SS-31 (elamipretide) representing the leading clinical candidate (Tung et al., 2025). Rather than relying primarily on ΔΨm, SS peptides accumulate through selective binding to cardiolipin, a signature phospholipid of the inner mitochondrial membrane (Chavez et al., 2020; Shin et al., 2024). This interaction stabilizes cristae architecture and modulates electron transport chain function. The dimethyltyrosine residue within SS-31 further confers direct antioxidant activity, suppressing cytochrome c peroxidase function and limiting cardiolipin oxidation, thereby preserving mitochondrial bioenergetics (Bai et al., 2021).

Other peptide-based strategies expand the functional scope of mitochondrial targeting. Mitochondria-penetrating peptides (MPPs), characterized by repeating hydrophobic and cationic residues, combine cell-penetrating and organelle-targeting properties and enable efficient mitochondrial delivery of small molecules with relatively low cytotoxicity (Abe et al., 2023; Schmitt and Wennemers, 2025). Hybrid constructs such as XJB peptides couple antioxidant moieties, including nitroxides, to mitochondrial membrane-affine peptide scaffolds derived from gramicidin S, thereby integrating targeting and ROS detoxification within a single architecture (Hara et al., 2024; Lu et al., 2025; Xun et al., 2012). In parallel, mitochondrial targeting sequences (MTS)—the N-terminal motifs that guide nuclear-encoded proteins through the TOM/TIM import machinery—have been adapted for protein engineering and gene therapy applications, providing a route for delivering macromolecular cargos directly to the mitochondrial matrix (Di Donfrancesco et al., 2022; Pfanner and Geissler, 2001).

In neurodegenerative disease models, MTPs consistently demonstrate neuroprotective activity. SS-31 reduces amyloid-*β*–induced mitochondrial damage and restores axonal mitochondrial transport in Alzheimer’s disease models, while in Parkinson’s disease it protects dopaminergic neurons and limits mitochondrial swelling in toxin-based paradigms (Reddy et al., 2017). Peptide inhibitors of pathological mitochondrial fission, such as P110, further illustrate how targeting mitochondrial dynamics can mitigate neuronal loss. In Huntington’s disease and amyotrophic lateral sclerosis models, peptide-based antioxidants—including XJB derivatives and SS-31—attenuate mitochondrial DNA damage, improve motor performance and extend survival in transgenic animals (Du et al., 2024; Rios et al., 2023).

Despite these advances, peptide strategies face translational challenges related to stability, membrane permeability and manufacturing cost. Proteolytic degradation and limited blood–brain barrier penetration remain major constraints for chronic neurodegenerative indications. Consequently, current research is increasingly integrating MTPs into nanocarrier platforms, including liposomes and polymeric systems, to enhance pharmacokinetics, protect peptide integrity and enable multimodal mitochondrial intervention. Compared with lipophilic cation strategies, SS peptides offer superior mitochondrial selectivity and tolerability and engage mitochondria through membrane potential–independent mechanisms; however, their limited blood–brain barrier permeability and susceptibility to proteolytic degradation remain the primary barriers to clinical translation for chronic neurodegenerative indications.

### 3.3 Other targeting ligands

Alongside lipophilic cations and peptide-based systems, mitochondrial targeting is increasingly explored through chemically and biologically diverse molecular strategies that extend beyond classical ligand design. These approaches draw on coordination chemistry, receptor-guided recognition and functional repair paradigms, reflecting an effort to overcome limitations associated with membrane potential–dependent accumulation, restricted cargo versatility and long-term safety (Boob et al., 2025; Liu Y. et al., 2022).

Coordination compounds represent a prominent direction within this diversification. Redox-active metal complexes based on manganese, ruthenium and iridium have been engineered to emulate endogenous antioxidant enzymes while retaining intrinsic mitochondrial affinity (Krasnovskaya et al., 2020; Liu B. et al., 2024). Manganese salen derivatives such as EUK-8 and EUK-134 exhibit combined superoxide dismutase and catalase activities, enabling catalytic suppression of mitochondrial oxidative stress (Chen L. et al., 2025; Jomova et al., 2023). Complementary strategies targeting metal homeostasis include mitochondria-directed iron chelators, such as hydroxylated coumarin derivatives exemplified by CT51, which mitigate iron-driven redox imbalance linked to neuronal vulnerability (Cilibrizzi et al., 2023). In parallel, cationic Ru and Ir complexes exploit their physicochemical properties to achieve mitochondrial localization, supporting applications ranging from mitochondrial DNA modulation to photodynamic intervention and imaging (Liu Z. et al., 2024; Prathima et al., 2023).

Targeting specificity can also be refined through receptor-mediated molecular recognition at the cellular interface. Ligands such as RVG29 facilitate neuronal engagement through acetylcholine receptor binding, whereas chlorotoxin and NCAM-mimetic peptides enhance neuronal uptake and can be combined with mitochondrial motifs to enable hierarchical targeting across cellular and subcellular levels (Han et al., 2021). These strategies illustrate how cell-type selectivity and organelle localization may be coordinated through molecular design rather than carrier architecture.

Additional progress arises from strategies that directly modulate mitochondrial integrity. Mitochondrial transplantation has demonstrated the capacity to restore respiratory function and attenuate apoptosis in preclinical models, while mitochondria-directed genome editing technologies are being explored to correct pathogenic mtDNA variants (Shoop et al., 2023). Meanwhile, several naturally mitochondriotropic molecules—including anthocyanins, melatonin, *α*-lipoic acid and idebenone—exhibit passive mitochondrial enrichment and provide metabolic or antioxidant support, although their targeting efficiency remains comparatively modest relative to engineered ligands (Chen X. et al., 2025).

Across mitochondrial targeting paradigms, trade-offs emerge between targeting efficiency, biological compatibility and functional breadth. Lipophilic cations enable robust mitochondrial accumulation through electrochemical driving forces but remain constrained by membrane potential dependence and concentration-sensitive toxicity (Klier et al., 2021; Lee et al., 2024). Peptide-based strategies partially alleviate these limitations by engaging lipid and protein interactions within mitochondrial membranes, supporting modulation of structural and dynamic processes, yet challenges related to stability and brain delivery persist (Zielonka et al., 2017). Emerging molecular approaches—including coordination complexes, receptor-guided ligands and biologically inspired repair strategies—expand targeting from electrochemical accumulation toward mechanistic intervention, positioning mitochondrial targeting as a multi-layered molecular design problem (Chen W. et al., 2023). Collectively, these advances establish a conceptual foundation in which diverse targeting ligands operate as complementary tools for addressing the heterogeneous mitochondrial pathology that characterizes neurodegenerative disease.

## 4 Carrier-based and nanotechnology-enabled mitochondrial delivery

### 4.1 Synthetic nanocarriers integrating mitochondrial ligands

Although molecular targeting ligands provide the biochemical basis for mitochondrial recognition, their therapeutic performance is often limited by insufficient cargo capacity, restricted pharmacokinetics and incomplete tissue specificity. These limitations have accelerated the development of carrier-based and nanotechnology-enabled delivery systems capable of integrating mitochondrial targeting with controlled transport, BBB penetration and multifunctional therapeutic modulation. The central role of mitochondria in cellular bioenergetics, apoptosis and redox signaling has positioned mitochondrial delivery as a critical objective in therapeutic development for neurodegenerative diseases (NDDs) (Choi et al., 2024). Achieving this objective, however, requires therapeutic cargos to traverse multiple biological barriers, including the blood–brain barrier (BBB), cellular membranes and the double-membrane architecture of mitochondria (Achar et al., 2021; Wu et al., 2023; Zha et al., 2024). Synthetic nanocarriers, typically ranging from 1 to 1,000 nm, offer a versatile platform to address these challenges by integrating mitochondrial-targeting ligands with controlled drug delivery architectures, thereby improving cargo stability, enhancing bioavailability, enabling spatial precision and reducing systemic toxicity (Beach et al., 2024; Sun et al., 2023).

Liposome-based systems represent one of the most mature carrier modalities for mitochondrial delivery (Sun et al., 2023). Their phospholipid bilayer structure allows simultaneous encapsulation of hydrophilic and hydrophobic therapeutics while providing a flexible surface for ligand conjugation. Incorporation of lipophilic cations such as TPP ^+^ or dequalinium enables electrophoretic mitochondrial accumulation, whereas additional targeting motifs—including the rabies virus glycoprotein–derived peptide RVG29—support neuronal uptake and BBB penetration (Han et al., 2021; Zielonka et al., 2017). Biomimetic strategies further extend liposomal functionality: membrane cloaking using erythrocyte or macrophage membranes prolongs circulation and facilitates immune evasion, while cargos such as curcumin, resveratrol and genistein delivered through these systems have demonstrated reduced amyloid burden, attenuated neuroinflammation and improved mitochondrial redox balance in Alzheimer’s disease models (Chen R. et al., 2023; Miao et al., 2022). More specialized constructs, exemplified by the MITO-Porter platform, exploit membrane fusion mechanisms to deliver macromolecules—including proteins and nucleic acids—directly into the mitochondrial matrix (Kumar et al., 2024).

Polymeric nanoparticles provide complementary advantages through tunable degradation kinetics, structural stability and modular surface chemistry. Conjugation strategies such as PLGA-b-PEG-TPP enable efficient mitochondrial enrichment while preserving controlled release profiles (Kuperkar et al., 2024). Increasingly, polymeric platforms incorporate environmental responsiveness; pH-sensitive nanoparticles, for instance, can exploit the mildly acidic microenvironment of injured brain tissue or dysfunctional mitochondria to trigger localized cargo release (Han et al., 2021; Singh and Nayak, 2023). Advanced multifunctional designs further illustrate the conceptual shift from delivery to intervention, integrating disease-sensing components—such as antibodies recognizing damaged mitochondria—with therapeutic payloads including siRNA targeting regulators of mitophagy, thereby enabling selective clearance of dysfunctional organelles (Sun et al., 2023; Zhai et al., 2022).

Smaller amphiphilic assemblies, including nanomicelles and dendritic polymers, offer high loading capacity for poorly soluble therapeutics and dense ligand presentation (Beach et al., 2024). Block-copolymer micelles modified with mitochondrial ligands and neuronal targeting peptides have been shown to restore synaptic function and rebalance mitochondrial dynamics in neurodegenerative models (Yang et al., 2020). Dendrimers such as PAMAM provide highly branched architectures with abundant functional groups for multivalent TPP conjugation, supporting efficient gene delivery and improved endosomal escape (Li et al., 2018).

Inorganic nanomaterials introduce additional physicochemical functionality to mitochondrial targeting strategies. Cerium oxide nanoparticles, particularly when modified with mitochondrial ligands, exhibit catalytic redox cycling between Ce^3+^ and Ce^4+^ states, enabling sustained reactive oxygen species scavenging and neuroprotection in models of Alzheimer’s and Parkinson’s disease (Jiang et al., 2024; Krug et al., 2025). Quantum dots functionalized with mitochondrial ligands enable simultaneous imaging and therapeutic modulation, while gold nanoparticles provide versatile scaffolds for ligand conjugation and controlled delivery of antioxidant or gene-based interventions (Guo et al., 2017; Liu F. et al., 2023).

Across these synthetic systems, several design principles emerge: appropriate surface charge to support mitochondrial tropism, sufficient cargo loading capacity, controllable release kinetics and minimal immunogenicity. Nonetheless, translational challenges remain, including potential membrane perturbation associated with cationic targeting motifs, incomplete understanding of *in vivo* clearance pathways and manufacturing complexity. As a result, synthetic nanocarriers are increasingly conceptualized not merely as transport vehicles but as adaptive platforms capable of sensing mitochondrial dysfunction and modulating disease-relevant pathways. This evolution marks a shift toward programmable mitochondrial intervention systems, positioning integrated nanocarriers as a central component of precision therapeutic strategies for neurodegenerative disorders.

### 4.2 Biomimetic and cell-derived mitochondrial delivery systems

Biomimetic mitochondrial delivery systems exploit natural biological components to overcome the physiological barriers that limit conventional targeting strategies. By incorporating cellular membranes or endogenous vesicular pathways, these platforms inherit immune evasion, prolonged circulation and improved tissue recognition, enabling more efficient delivery to neuronal mitochondria across the blood–brain barrier (Chen et al., 2022; Lv et al., 2025; Zou et al., 2023).

Cell membrane–coated nanoparticles represent a prominent approach. Membranes derived from macrophages or erythrocytes can camouflage synthetic carriers, reduce clearance and facilitate hierarchical targeting when combined with mitochondrial ligands such as triphenylphosphonium and neuronal recognition peptides (Duan et al., 2023; Zhu et al., 2022). In neurodegenerative disease models, these systems enhance mitochondrial delivery of antioxidants and metabolic modulators, leading to reduced oxidative stress and improved neuronal function (Boccardi et al., 2025; Qian et al., 2022).

Extracellular vesicles provide an endogenous delivery modality with inherent barrier-crossing capability. Mitochondria-derived vesicles, in particular, participate in mitochondrial quality control by trafficking damaged components toward degradation pathways, linking vesicular transport with regulation of neuroinflammation and mitochondrial homeostasis (Prashar et al., 2024; Rosina et al., 2022; Todkar et al., 2021).

At a more integrative level, mitochondrial transplantation and intercellular mitochondrial transfer aim to directly restore organelle function. Transfer of functional mitochondria from supporting cells to injured neurons has been shown to recover bioenergetics, reduce oxidative stress and slow neurodegeneration in preclinical models, while alternative administration routes such as intranasal delivery suggest translational feasibility (Liu et al., 2021; Liu Z. et al., 2022).

Although challenges remain—including scalability, standardization and long-term safety—biomimetic and cell-derived strategies reflect a conceptual shift from molecular targeting toward organelle repair, positioning these platforms as an emerging frontier in mitochondrial therapy for neurodegenerative disease. Nevertheless, critical limitations in this area remain insufficiently discussed. Batch-to-batch reproducibility is a persistent concern for cell-derived systems, as donor-dependent variability in membrane composition, surface protein expression and cargo loading efficiency introduces inconsistency that complicates preclinical-to-clinical translation. Immune compatibility also remains incompletely characterized: although autologous cell-derived vesicles theoretically minimize immunogenicity, allogeneic sources introduce unpredictable immune responses that have not been systematically evaluated in neurodegeneration-relevant *in vivo* models. Furthermore, no consensus manufacturing standards currently exist for biomimetic mitochondrial delivery platforms, limiting cross-study comparability and regulatory pathway clarity. Addressing these gaps is a prerequisite for advancing biomimetic strategies beyond proof-of-principle demonstrations.

### 4.3 Stimuli-responsive and multimodal mitochondrial delivery

To enhance precision while minimizing off-target effects, mitochondrial delivery strategies are increasingly designed to respond dynamically to disease-associated cues. Stimuli-responsive platforms exploit features of the neurodegenerative microenvironment—such as altered pH, elevated ROS and external physical triggers—to enable spatially and temporally controlled therapeutic activation (Ballance et al., 2019; Dai et al., 2025). At the same time, the integration of imaging, diagnostic and therapeutic functions have positioned multimodal mitochondrial delivery as a central direction in next-generation neurotherapeutics (Jia et al., 2018).

pH-responsive systems represent one of the most established approaches. Because injured neuronal regions and intracellular endolysosomal compartments exhibit mild acidification, carriers incorporating acid-labile bonds or charge-reversal chemistries can undergo structural transformation that promotes endosomal escape and subsequent mitochondrial localization. Such designs allow selective release of antioxidants or metabolic modulators within diseased tissue while limiting exposure to healthy cells (Meng J.-L. et al., 2025).

ROS-responsive strategies leverage mitochondrial oxidative stress as an intrinsic activation signal. Incorporation of ROS-cleavable linkers enables drug release specifically within dysfunctional mitochondria, coupling therapeutic activation to pathological burden. This mechanism introduces a feedback element in which ROS both triggers and is attenuated by treatment, enhancing subcellular specificity and therapeutic efficiency (Saravanakumar et al., 2017; W. Zhang et al., 2019).

External stimulus–guided delivery further expands functional control. Photoresponsive systems combining mitochondrial ligands with photosensitizers enable light-triggered modulation of mitochondrial processes, including protein aggregation, membrane potential restoration and controlled drug release (Xiao et al., 2025; Zhang et al., 2019). More broadly, photobiomodulation and emerging mitochondrial optogenetic approaches illustrate how mitochondrial activity itself can be regulated with high spatiotemporal precision, moving beyond passive targeting toward active organelle modulation (Lee S.-Y. et al., 2023).

Multimodal platforms integrate these principles within hierarchical delivery architectures. By combining brain-targeting ligands with mitochondrial targeting motifs, carriers can sequentially traverse physiological barriers and localize to neuronal mitochondria (Hara et al., 2024). Incorporation of sensing modules enables real-time monitoring of mitochondrial biomarkers such as ATP or glutathione, while therapeutic cargos simultaneously address oxidative stress, proteostasis and mitochondrial quality control (Yang, 2025). Such theranostic systems reflect a shift toward coordinated intervention across multiple nodes of neurodegenerative pathology.

Despite these advances, challenges remain. Stimulus-responsive systems must balance sensitivity with stability, avoid excessive cation-associated toxicity and demonstrate predictable pharmacokinetics within the central nervous system. Nevertheless, the convergence of responsive chemistry, multimodal integration and organelle-level regulation indicates a broader evolution in mitochondrial medicine—from static targeting toward adaptive, context-aware therapeutic frameworks capable of addressing the heterogeneous progression of neurodegenerative disease.

## 5 Mitochondria-targeted therapeutic modalities

Advances in mitochondrial targeting have progressively shifted the therapeutic landscape of neurodegenerative disease from symptomatic modulation toward organelle-level intervention (Yang, 2025). Early strategies largely focused on mitigating oxidative stress, yet growing mechanistic insight now reveals mitochondrial dysfunction as a systems-level process encompassing bioenergetic failure, impaired quality control, disrupted signalling and inflammatory activation (Choo et al., 2004). Consequently, mitochondria-targeted therapeutics are evolving from single-function agents into integrated modalities that simultaneously restore redox balance, reprogram metabolism, regulate mitochondrial dynamics and enable molecular repair. This conceptual transition reframes delivery not merely as an enabling step but as a determinant of therapeutic mechanism, positioning mitochondrial intervention as a unifying framework linking cellular pathology to disease modification across neurodegenerative disorders.

### 5.1 Redox and bioenergetic restoration

Mitochondrial oxidative stress and bioenergetic decline represent early and converging drivers of neurodegeneration, positioning restoration of mitochondrial redox balance and energy metabolism as a central therapeutic objective (Bhatti et al., 2022; Cenini et al., 2019; Wasim, 2025). Conventional antioxidants are limited by poor subcellular delivery; by contrast, mitochondria-targeted strategies enable localized modulation of ROS and respiratory function (Martinelli et al., 2020). Quinone-based lipophilic cation conjugates such as MitoQ and SkQ1 exemplify this approach, coupling mitochondrial enrichment with reversible redox cycling to attenuate oxidative damage and stabilize electron transport chain activity (Lyamzaev et al., 2023; Rondeau et al., 2024). Peptide-based systems, including SS-31 (elamipretide), operate through cardiolipin binding to preserve cristae architecture and limit cytochrome c–mediated oxidative reactions, thereby supporting ATP synthesis (Yang et al., 2020). Complementary catalytic antioxidants, such as nitroxide derivatives (for example MitoTEMPO) and metal enzyme mimetics including manganese salen complexes, extend redox buffering capacity by detoxifying superoxide and hydrogen peroxide within mitochondria (Grujicic and Allen, 2024).

Because oxidative stress and energetic failure are tightly coupled, therapeutic strategies increasingly aim to restore metabolic resilience alongside ROS control. Modulation of NAD^+^ homeostasis using precursor supplementation enhances respiratory efficiency and activates protective sirtuin signaling, whereas metabolic modulators and transcriptional regulators—including AMPK–PGC-1α and Nrf2 pathways—promote mitochondrial biogenesis and functional recovery (Katsyuba et al., 2018). Buffering cellular energy stores through phosphocreatine systems provides an additional layer of protection in metabolically vulnerable neurons (Srivastava, 2016).

Perturbations in mitochondrial metal homeostasis further link oxidative stress to neuronal injury. Excess labile iron amplifies ROS generation via Fenton chemistry, and mitochondria-directed chelators have therefore emerged as adjunct strategies to interrupt this cycle and preserve neuronal circuitry (Aguirre et al., 2017). Collectively, these approaches highlight a conceptual shift from isolated antioxidant intervention toward integrated restoration of mitochondrial redox control, metabolic capacity and organelle resilience in neurodegenerative disease.

### 5.2 Mitochondrial quality control and network remodeling

Mitochondrial quality control (MQC) encompasses a coordinated network of processes that maintain mitochondrial integrity, including dynamics (fusion and fission), biogenesis and mitophagy (Xian and Liou, 2021). In neurodegenerative disorders, disruption of this balance leads to the accumulation of dysfunctional mitochondria, impaired energy supply and heightened neuronal vulnerability. Therapeutic strategies therefore increasingly aim to restore MQC as a systems-level approach to rebuilding neuronal mitochondrial networks (Fields et al., 2023; Zhang et al., 2025).

Modulation of mitochondrial dynamics represents one of the most direct intervention points. Excessive fission, driven by hyperactivation of regulators such as Drp1, contributes to mitochondrial fragmentation and synaptic dysfunction (Sabouny and Shutt, 2020; Tábara et al., 2025; Xi et al., 2025). Pharmacological inhibition of Drp1, including small molecules such as Mdivi-1 or peptide inhibitors like P110 that disrupt Drp1–Fis1 interactions, reduces fragmentation and preserves neuronal viability in multiple disease models (Johnson et al., 2021; Parida et al., 2023; Rios et al., 2023). Conversely, approaches that promote fusion—through upregulation of mitofusins or stabilization of inner membrane architecture—support functional complementation within mitochondrial populations. Agents originally developed as mitochondrial antioxidants, including MitoQ and SS-31, illustrate how redox modulation can secondarily restore mitochondrial morphology and network connectivity (Fanibunda et al., 2019; Kang et al., 2020; Li et al., 2022).

Complementary strategies focus on mitochondrial biogenesis, a process largely governed by the PGC-1α transcriptional axis (Jamwal et al., 2021). Reduced PGC-1α signaling is a common feature across neurodegenerative diseases and contributes to diminished mitochondrial renewal (Chen J. et al., 2025; Suntar et al., 2020). Pharmacological activation of PPAR pathways, modulation of AMPK–SIRT1 signaling and supplementation of NAD^+^ precursors have each been shown to enhance mitochondrial biogenesis, improving ATP production and metabolic resilience in preclinical models (Yap et al., 2020).

Selective elimination of damaged mitochondria through mitophagy represents the third pillar of MQC restoration. The PINK1/Parkin pathway coordinates ubiquitination and autophagic clearance of dysfunctional organelles, and its impairment is strongly linked to neurodegeneration (Agarwal and Muqit, 2022; Tu et al., 2022). Small molecules that stabilize PINK1 signaling, natural compounds such as urolithin A that stimulate mitophagy, and inhibitors of negative regulators including USP30 collectively demonstrate that enhancing mitochondrial turnover can reduce oxidative stress and proteotoxic burden (Kazi et al., 2025; Wen et al., 2021).

Beyond pharmacological modulation, emerging interventions seek to directly reshape mitochondrial networks. Strategies that restore axonal mitochondrial trafficking improve energy distribution at synapses, while approaches such as mitochondrial transplantation highlight the possibility of rapidly reconstituting bioenergetic capacity in severely compromised neurons (Han et al., 2020; Li J. et al., 2021). Together, these advances support a unifying therapeutic concept in which coordinated regulation of dynamics, biogenesis and mitophagy enables systemic reconstruction of neuronal mitochondrial function. Future efforts are likely to prioritize multi-node interventions capable of synchronizing these processes, reflecting the networked nature of mitochondrial pathology in neurodegenerative disease.

### 5.3 Organelle replacement and genetic correction

As mitochondrial damage becomes irreversible during disease progression, therapeutic strategies are increasingly shifting from functional modulation toward direct restoration of mitochondrial integrity (Li et al., 2025). Two emerging approaches—mitochondrial transplantation and mitochondrial genome editing—aim to repair bioenergetic failure at its structural and genetic origins (Choo et al., 2004).

Mitochondrial transplantation, often referred to as mitotherapy, involves the delivery of functional mitochondria into compromised cells to restore respiratory capacity and cell survival. Intercellular mitochondrial transfer is now recognized as a physiological process mediated by tunneling nanotubes, extracellular vesicles and cell fusion, providing a biological rationale for therapeutic application (de Assis Fernandes Caldeira et al., 2025; Kubat et al., 2025). Experimental studies demonstrate that astrocyte-derived mitochondria can be transferred to injured neurons, improving ATP production and reducing apoptosis. In neurodegenerative models, administration of isolated mitochondria—via systemic, intracerebral or intranasal routes—has been shown to enhance respiratory chain activity, preserve neuronal populations and improve behavioral outcomes. Biomimetic platforms, including exosome-assisted mitochondrial delivery, further suggest feasible strategies for increasing stability and reducing immunogenicity (Eo et al., 2024; Hayakawa et al., 2016; Kim J. S. et al., 2023).

Parallel efforts focus on correcting pathogenic mtDNA alterations. Because mtDNA mutations accumulate under oxidative stress and contribute directly to respiratory dysfunction, targeted genome editing offers a route to durable disease modification. Mitochondria-targeted nucleases such as mitoZFNs and mitoTALENs enable selective removal of mutant mtDNA, thereby shifting heteroplasmy toward functional genomes (Gammage et al., 2018; Shoop et al., 2023; Silva-Pinheiro et al., 2023). More recently, CRISPR-independent base editors, including DdCBE, have enabled precise nucleotide conversion within mtDNA, expanding the scope of mitochondrial gene therapy (Lee S. et al., 2023; Mok et al., 2020). Although delivery of large editing complexes into mitochondria remains technically challenging, advances in targeting sequences, RNA import strategies and viral vectors are beginning to overcome these barriers (Raguram et al., 2022; Tung et al., 2025).

Despite their conceptual promise, organelle replacement and genetic correction face substantial translational hurdles, including efficient targeting of affected neuronal populations, long-term safety, immune compatibility and scalable delivery. Nevertheless, these approaches mark a fundamental transition in mitochondrial therapeutics—from compensating for dysfunction to directly reconstructing organelle health—highlighting the potential for disease-modifying interventions in neurodegenerative disorders.

However, several specific limitations and unresolved questions in this space warrant explicit acknowledgement. For mitochondrial transplantation, it remains mechanistically unclear whether transplanted organelles are stably integrated into recipient neurons, retained over biologically meaningful timescales, and capable of sustaining respiratory function *in vivo*; most evidence derives from short-term experiments in simplified cellular or animal models. For genome editing, the efficiency of delivering large editing complexes—including mitoTALEN and DdCBE—into mitochondria across relevant neuronal populations remains a fundamental bottleneck, and the long-term safety profile of persistent mitochondrial genome modification, including off-target editing risk, has not been adequately characterized. Both strategies also lack robust disease-stage-specific efficacy data, leaving open the question of when during disease progression these interventions are most likely to provide meaningful benefit. These unresolved questions, alongside the broader translational challenges that span all therapeutic modalities reviewed, define the key priorities for the field going forward—the subject of the following section.

## 6 Challenges and outlook

Systematic comparison of the delivery and targeting strategies discussed above reveals distinct translational trade-offs across efficacy, safety, manufacturability, and clinical feasibility (Table 3). Lipophilic cation–based systems, exemplified by TPP^+^ conjugates, consistently achieve high levels of mitochondrial accumulation; however, their performance remains strongly dependent on mitochondrial membrane potential and is frequently limited by dose-dependent toxicity at therapeutically relevant exposures. Peptide-based platforms such as SS-31 exhibit improved biocompatibility and relative membrane potential independence, yet their therapeutic translation is hindered by inefficient blood–brain barrier penetration in the absence of auxiliary delivery strategies. Nanocarrier formulations provide substantially greater flexibility in cargo loading and multifunctional integration, although challenges associated with large-scale manufacturing, batch reproducibility, pharmacokinetic heterogeneity, and long-term clearance remain insufficiently resolved. Biomimetic and cell-derived systems offer inherent immunocompatibility and biological adaptability, but their broader clinical implementation is constrained by limited standardization and manufacturing complexity. At the organelle level, emerging approaches including mitochondrial transplantation and mitochondrial genome editing directly address the underlying basis of mitochondrial dysfunction, yet currently face the most substantial translational obstacles, particularly with respect to delivery efficiency, immunogenicity, and reproducibility. Collectively, these comparisons emphasize that no single platform is likely to provide a universally effective solution for mitochondrial targeting. Instead, future progress will likely depend on therapeutics tailored to disease stage, tissue context, and patient-specific pathological heterogeneity.

**Table 3:** Comparative overview of mitochondrial targeting strategies for neurodegenerative diseases.

Strategy category	Representative agents/platforms	BBB penetration	Mitochondrial selectivity	Dose-limiting toxicity	Clinical stage	Reproducibility/scalability	Primary translational bottlenecks
Lipophilic Cations (TPP^+^ conjugates)	MitoQ (Murphy and Smith, 2007; Smith et al., 2003), SkQ1 (Skulachev et al., 2009), MitoTEMPO (Dikalov and Harrison, 2014; Trnka et al., 2008), MitoVitE (McCormick et al., 2016; Plecitá-Hlavatá et al., 2009)	Moderate	High	High at elevated conc. (membrane disruption)	Phase II (MitoQ in PD/AD)	Good	ΔΨm dependence; chronic safety; restricted cargo range
Szeto–Schiller Peptides (SS peptides)	SS-31/Elamipretide (Tung et al., 2025; Mitchell et al., 2020; Du et al., 2024), SS-20 (Chavez et al., 2020)	Low–Moderate	High	Low; generally well-tolerated	Phase II (cardiac/renal; limited NDD data)	Moderate	BBB penetration; proteolytic degradation; NDD trial data limited
Mitochondria-Penetrating Peptides (MPPs)	XJB peptides (Xun et al., 2012; Hara et al., 2024), P110 (Rios et al., 2023), custom MPP constructs (Abe et al., 2023; Schmitt and Wennemers, 2025)	Low–Moderate	Moderate–High	Moderate; sequence-dependent	Preclinical	Moderate	Stability; synthesis cost; *in vivo* BBB data lacking
Synthetic Nanocarriers (liposomes, polymeric NPs, dendrimers)	TPP^+^-liposomes (Sun et al., 2023; Zielonka et al., 2017), PLGA-b-PEG-TPP (Kuperkar et al., 2024), PAMAM dendrimers (Li et al., 2018), MITO-Porter (Kumar et al., 2024)	Moderate–High	Moderate	Variable; cationic motifs may perturb membranes	Preclinical (select Phase I)	Low–Moderate	Batch reproducibility; endosomal escape; *in vivo* clearance; scale-up
Biomimetic & Cell-Derived Systems (membrane-coated NPs, EVs)	Macrophage/RBC membrane-coated NPs (Han et al., 2021; Chen W. et al., 2023), exosomes (Eo et al., 2024; Jia et al., 2018), mitochondria-derived vesicles (Hayakawa et al., 2016; Kim J. et al., 2023)	High	Moderate	Low (favorable immunocompatibility)	Preclinical	Low	Standardization; manufacturing scalability; immune variability across donors
Stimuli-Responsive Platforms (pH/ROS/photo-triggered)	ROS-cleavable nanocarriers (Saravanakumar et al., 2017; Zhang et al., 2019), pH-sensitive polymers (Meng J.-L. et al., 2025; Han et al., 2021), photoresponsive systems (Xiao et al., 2025; Lee S.-Y. et al., 2023)	Moderate	Moderate–High	Moderate; activation specificity critical	Preclinical	Low–Moderate	CNS pharmacokinetics; stimulus sensitivity vs. stability trade-off; phototoxicity risk
Coordination Compounds & Receptor-Guided Ligands	EUK-134/Mn-salen (Chen J. et al., 2025; Jomova et al., 2023), Ru/Ir complexes (Krasnovskaya et al., 2020; Prathima et al., 2023), RVG29 (Han et al., 2021), CT51 (Cilibrizzi et al., 2023)	Low–Moderate	Moderate	Variable; metal toxicity a concern	Preclinical	Moderate	Targeting specificity; metal clearance safety; limited *in vivo* NDD validation
Mitochondrial Transplantation (Mitotherapy)	Isolated mitochondria via intracerebral/intranasal/IV (Kubat et al., 2025; Liu et al., 2021; Hayakawa et al., 2016), exosome-assisted delivery (Eo et al., 2024; de Assis Fernandes Caldeira et al., 2025)	Variable	High	Immunogenicity risk; donor variability	Preclinical (early clinical in cardiac)	Low	Scalability; stability of isolated mitochondria; immune compatibility; delivery route optimization
Mitochondrial genome editing	mitoZFN (Shoop et al., 2023), mitoTALEN (Gammage et al., 2018; Silva-Pinheiro et al., 2023), DdCBE/CRISPR-independent base editor (Mok et al., 2020; Lee S.-Y. et al., 2023; Raguram et al., 2022)	Low	High	Off-target editing; delivery vector toxicity	Preclinical	Low	Delivery of large editing complexes into mitochondria; off-target risk; long-term safety; limited NDD models

**Figure 6:**
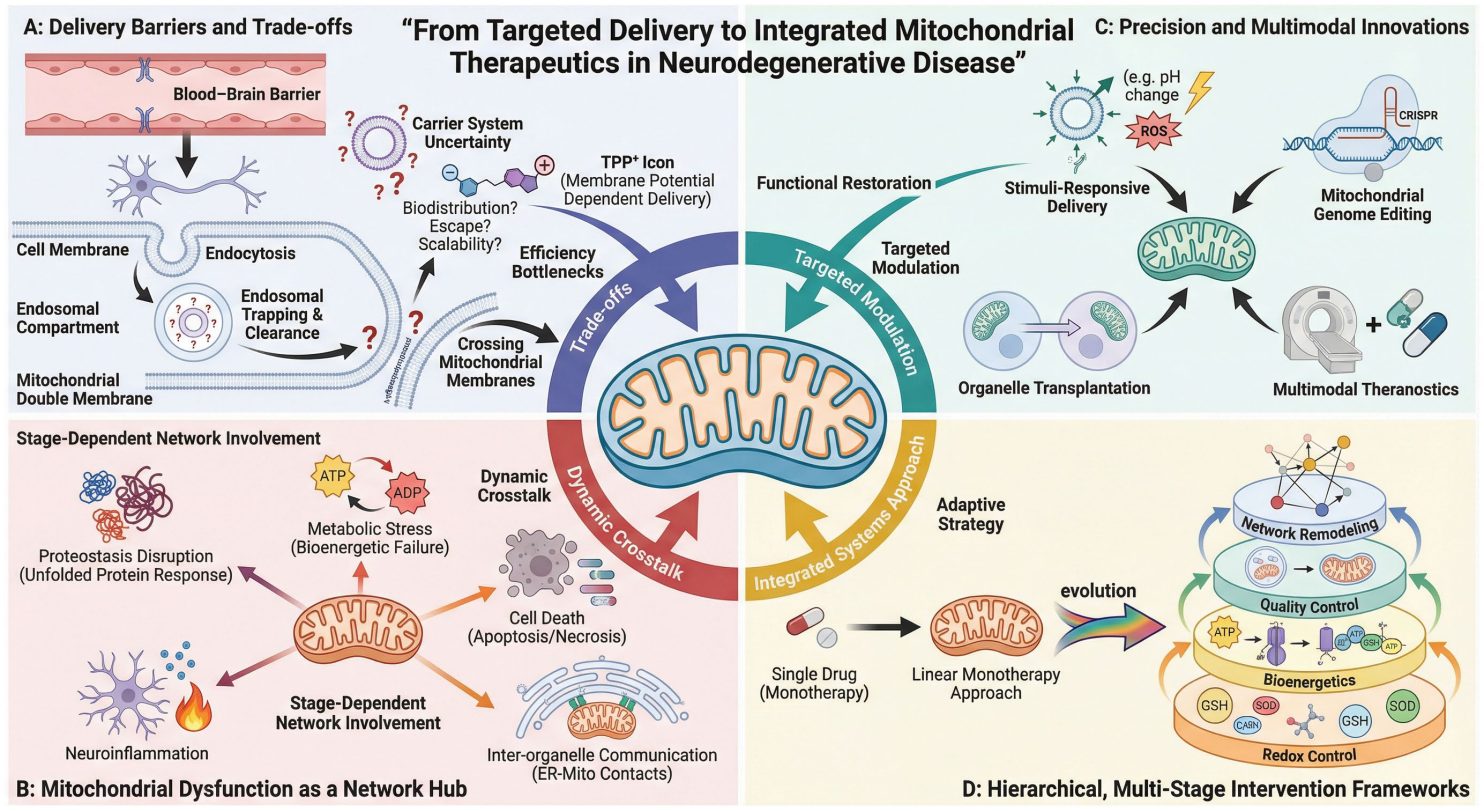
Conceptual evolution of mitochondrial targeting toward integrated therapeutic frameworks in neurodegenerative disease. Mitochondria function as central regulatory hubs linking metabolic homeostasis, proteostasis, inflammatory signalling and neuronal survival, and therefore represent key intervention points across neurodegenerative disorders. **(A)** Delivery barriers. Effective mitochondrial therapeutics must traverse multiple biological layers, including the blood–brain barrier, cellular membranes, endosomal compartments and the mitochondrial double membrane. Ligand-based strategies such as TPP^+^ highlight the balance between efficient electrochemical accumulation and potential bioenergetic perturbation, whereas carrier-enabled delivery expands cargo scope but introduces challenges related to biodistribution, intracellular trafficking and long-term clearance. **(B)** Network pathology. Mitochondrial dysfunction operates as a dynamic systems-level disturbance that integrates impaired protein homeostasis, metabolic stress, oxidative damage, neuroinflammation, disrupted inter-organelle communication and apoptotic signalling, with stage-dependent effects across disease progression. **(C)** Emerging precision interventions. Stimuli-responsive delivery systems, mitochondrial genome editing, organelle transplantation and multimodal theranostic platforms aim to directly restore mitochondrial function while enabling spatiotemporal targeting and real-time monitoring. **(D)** Paradigm transition. Converging advances in ligand chemistry, peptide engineering, carrier design and biologically inspired therapies support a shift from single-component targeting toward hierarchical, multi-layered strategies that coordinate redox control, bioenergetics, mitochondrial quality maintenance and network remodeling. Together, these developments position mitochondrial targeting as a conceptual foundation for disease-modifying therapy rather than solely a delivery approach.

Despite rapid advances in mitochondrial targeting, translating these strategies into effective therapies for neurodegenerative disease remains a substantial challenge. Therapeutic agents must traverse multiple biological barriers—including the blood–brain barrier, cellular membranes and the double-membrane architecture of mitochondria—while maintaining precise spatiotemporal activity. Widely used lipophilic cations such as TPP^+^ exemplify this tension: although highly efficient for mitochondrial accumulation, their membrane potential dependence can bias delivery toward relatively polarized organelles and excessive enrichment may perturb oxidative phosphorylation. Carrier-based approaches expand cargo versatility but introduce additional uncertainties related to biodistribution, endosomal escape, long-term clearance and manufacturing scalability (Figure 6A).

These translational constraints are compounded by the biology of neurodegeneration itself. Clinical manifestation typically follows extensive neuronal loss, narrowing the therapeutic window, while disease heterogeneity limits the impact of single-mechanism interventions (Figure 6B). Moreover, experimental models only partially capture the temporal and cellular complexity of human pathology, contributing to the variable outcomes observed in clinical trials of mitochondrial therapeutics. Together, these factors underscore that mitochondrial dysfunction operates not as an isolated lesion but as a dynamic network disturbance integrating proteostasis, metabolism, inflammation and inter-organelle communication. It should be noted that the therapeutic concepts discussed herein—including mitochondria-targeted drug delivery, organelle transplantation and genome editing—remain predominantly preclinical, and the translation of these strategies into clinically validated disease-modifying therapies will require rigorous demonstration of safety, efficacy and reproducibility in human studies.

Future progress will therefore depend on precision and integration rather than incremental improvements in targeting efficiency alone (Figure 6C). Stimuli-responsive delivery, mitochondrial genome editing and organelle transplantation point toward strategies capable of restoring mitochondrial function directly, while multimodal platforms that combine targeting, imaging and therapy may enable real-time monitoring of mitochondrial states and patient stratification. More broadly, advances in ligand chemistry, peptide engineering and biologically inspired delivery are converging toward hierarchical therapeutic frameworks that coordinate redox control, bioenergetics, quality maintenance and network remodeling across disease stages.

As chemical biology, nanomedicine and mitochondrial genetics continue to intersect, mitochondrial targeting is shifting from a delivery solution to a conceptual foundation for neurodegenerative therapy (Figure 6D). Harnessing this transition will be essential for moving beyond symptomatic mitigation toward durable, disease-modifying interventions.
